# Universal Theory of Light Scattering of Randomly Oriented Particles: A Fluctuational-Electrodynamics Approach for Light Transport Modeling in Disordered Nanostructures

**DOI:** 10.1021/acsphotonics.1c01710

**Published:** 2022-02-04

**Authors:** Francisco V. Ramirez-Cuevas, Kargal L. Gurunatha, Ivan P. Parkin, Ioannnis Papakonstantinou

**Affiliations:** † Photonic Innovations Lab, Department of Electronic and Electrical Engineering, University College London, London, WC1E 7JE, United Kingdom; ‡ Center for Energy Transition, Facultad de Ingeniería y Ciencias, Universidad Adolfo Ibáñez, Santiago, 7941169, Chile; § Department of Chemistry, University College London, London, WC1H 0AJ, United Kingdom

**Keywords:** fluctuational electrodynamics, random media, multiple scattering, Monte Carlo, computational nanophotonics

## Abstract

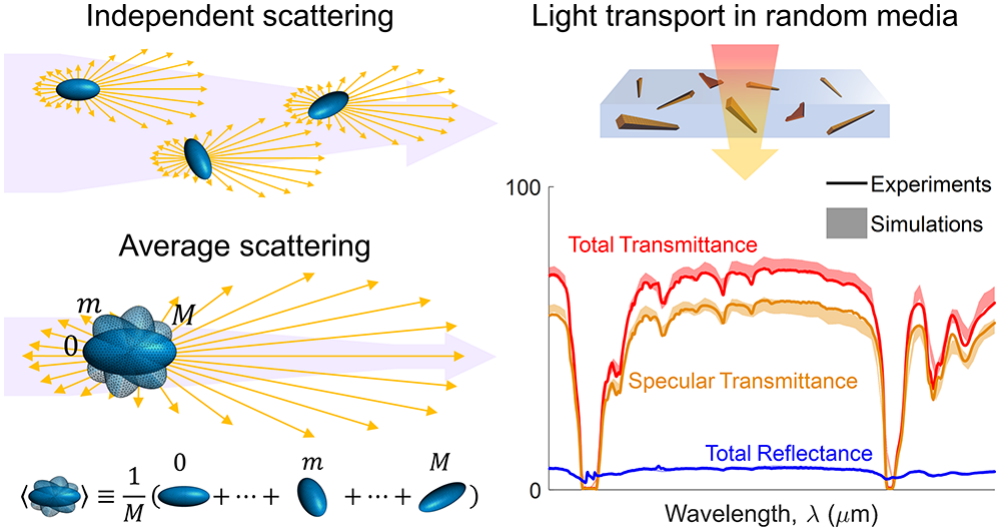

Disordered nanostructures are commonly encountered in many nanophotonic systems, from colloid dispersions for sensing to heterostructured photocatalysts. Randomness, however, imposes severe challenges for nanophotonics modeling, often constrained by the irregular geometry of the scatterers involved or the stochastic nature of the problem itself. In this Article, we resolve this conundrum by presenting a universal theory of averaged light scattering of randomly oriented objects. Specifically, we derive expansion-basis-independent formulas of the orientation-and-polarization-averaged absorption cross section, scattering cross section, and asymmetry parameter, for single or a collection of objects of arbitrary shape. These three parameters can be directly integrated into traditional unpolarized radiative energy transfer modeling, enabling a practical tool to predict multiple scattering and light transport in disordered nanostructured materials. Notably, the formulas of average light scattering can be derived under the principles of fluctuational electrodynamics, allowing the analogous mathematical treatment to the methods used in thermal radiation, nonequilibrium electromagnetic forces, and other associated phenomena. The proposed modeling framework is validated against optical measurements of polymer composite films with metal-oxide microcrystals. Our work may contribute to a better understanding of light–matter interactions in disordered systems, such as plasmonics for sensing and photothermal therapy, photocatalysts for water splitting and CO_2_ dissociation, photonic glasses for artificial structural colors, and diffuse reflectors for radiative cooling, to name just a few.

Predicting the complex optical phenomena manifesting in disordered nanomaterials represents a major challenge in the field of computational modeling. Plasmonic nanoparticle dispersions for sensing and photothermal therapy,^1,2^ heterostructured photocatalysts for water splitting and CO_2_ dissociation,^3^ diffuse reflectors for radiative cooling,^4^ and porous membranes for solar water desalination^5^ are a few examples where the complex inter-relation between near-field coupling and multiple scattering with a variation in particle morphology, orientation, and size impose severe limitations to theoretically predict the system optical response. Conventional modeling based on computational electromagnetics, such as the Finite Difference or Finite Elements methods, are often unsuitable to quantify the macroscopic optical properties of random media, namely, their specular and total transmittance/reflectance or the intensity distribution, all critical to assess the performance of these systems. Stochastic methods appear as the most appropriate alternative to calculate these properties, yet, with few exceptions, their applicability is limited to composite media containing subwavelength structures (effective media approximations)^6^ or spherical particles (radiative transfer simulations).^7^ As a result of the limitations in modeling, the majority of designs in disordered nanophotonic materials are driven by a phenomenological approach, whereby multiple samples are fabricated and tested in an iterative process that is time-consuming and expensive.

Light transport in random media is commonly addressed through the radiative transfer theory,^8,9^ which describes the propagation of the light specific intensity through a composite medium containing a random distribution of independently scattering particles. It is notable that, in principle, the theory is applicable to arbitrary particle geometries and groups of particles.^9^ In practice, however, radiative transfer modeling is most commonly applied to study light transport in composites with spherical particles.^7,10^ This is due to the scattering properties of the spherical particles being independent of the direction and polarization of the incident light.^11^ In this particular scenario, the solution of the radiative transfer equation (RTE) for unpolarized light requires three parameters: the particle’s absorption cross section, *C*_abs_, the scattering cross section, *C*_sca_, and the asymmetry parameter, μ_sca_.^7,12^ The latter is an indicator of the scattering anisotropy and a key element to calculate the angular distribution of scattered fields.^12^

The scattering of nonspherical particles, on the other hand, varies with the incident angle and polarization, and the RTE usually becomes too complex to solve.^9^ Under the independent scattering approximation, however, the correlation of the scattered field from different particles vanishes,^9^ and the scattering properties of an ensemble of randomly oriented particles can be approximated by the orientation averaged from a single particle (Figure 1a). For unpolarized light, the RTE becomes scalar,^14^ and the orientation and polarization averaged *C*_abs_, *C*_sca_, and μ_sca_ parameter triad can be used for radiative transfer simulations of arbitrary particles, following the same methodology of spherical particles. The same principle could be applied to more complex scenarios, for instance, a medium containing denser particle distributions or even particle clusters. In this case, the averaging should now be performed over a properly chosen collection of particles for which the effects from short-range correlations (due to collective interaction and interference of scattered fields) are prevalent (Figure 1b).^13^ Similarly, heterogeneous systems with particles of different sizes and optical properties can also be studied.

**Figure 1:**
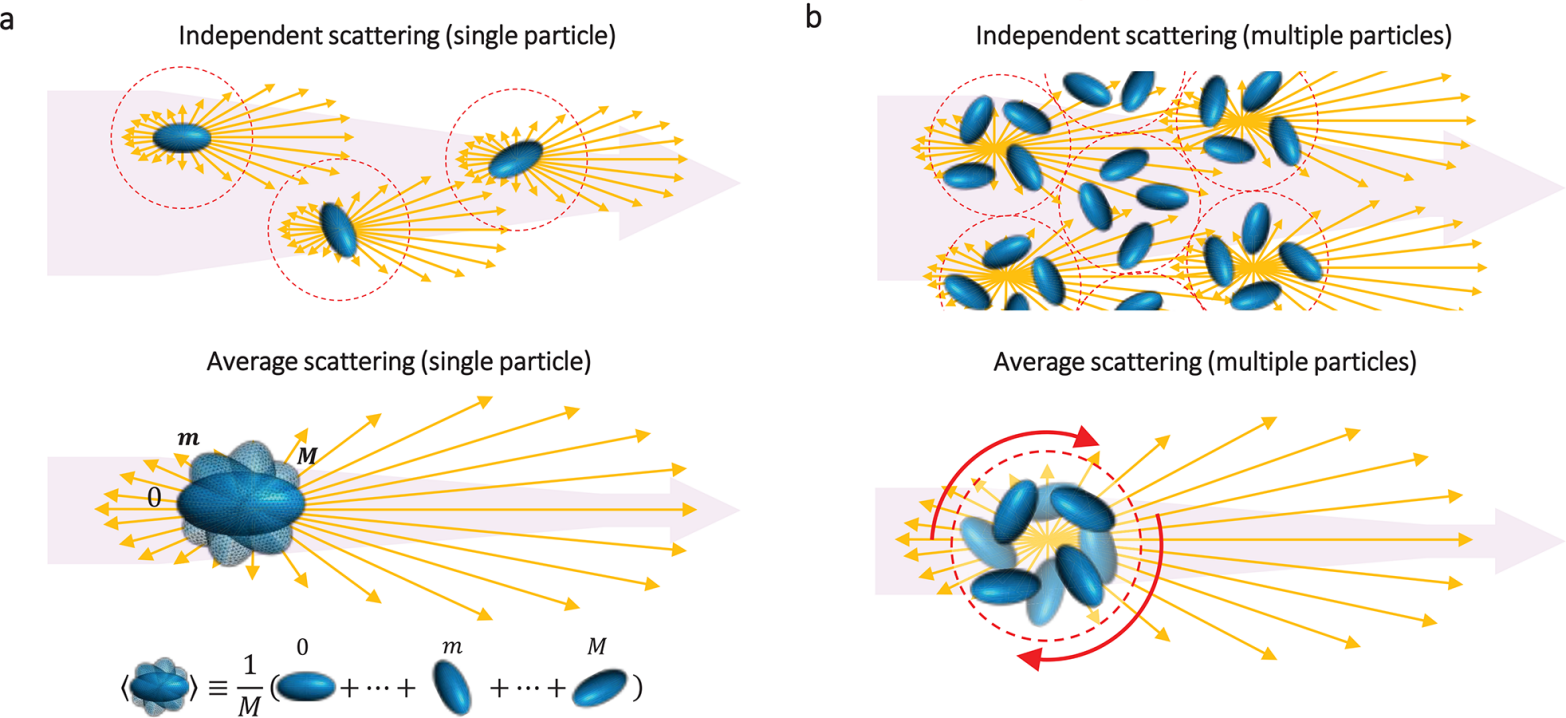
Average scattering of randomly oriented particles. (a) A collection of independent scattering particles (in this case prolate spheroids) randomly oriented in space, is approximated by the averaged sum over all possible particle’s orientations. In the schematic at the bottom, the index *m* represents a particular orientation of the particle, and *M* is the total number of particle orientations. In both schematics, the intensity of the incident light beam (purple arrow) decays due to scattering of the particle (yellow arrows). The red dotted circle represents a characteristic domain where short-range correlations are relevant. (b) In more dense particle systems, the independent scattering approximation applies to a collection of particles within a properly chosen domain that considers the effects of short-range correlations.^13^ Average scattering, thus, is calculated over a characteristic collection of particles.

The computation of orientation and polarization average scattering (average scattering, from now onward) for arbitrary nanoparticles is, however, nontrivial. Standard brute-force methods based on averaging over many plane-wave simulations at different angles of incidence and polarizations can be computationally expensive.^15^ Alternatively, semianalytical solutions relying on spherical wave expansion have demonstrated considerable improvements in the efficiency of the calculations.^15−19^ For example, formulas for direct computation of average scattering have been developed for axially symmetric objects, such as cylinders,^16^ spheroids,^16^ and clusters of spherical particles.^17^ However, the restrictive use of the spherical wave basis in this approach still imposes some constraints. Such is the case for objects with no axial symmetry or with sharp edges where the expansion of the scattered fields into spherical waves is not trivial.^16^ Often, in these problems, the scattered fields are more conveniently expanded through the basis relying on surface or volume discretization, such as surface currents in the Boundary Elements Method^20^ (BEM) or discrete dipoles in the DDA.^21^ For average scattering calculations, nonetheless, the expanded fields have to be transformed into spherical waves, and the efficiency of the method is appreciably reduced.^21^

In this Article, we present a universal theory of average light scattering from randomly oriented scatterers (single or collections of objects) of arbitrary shape and demonstrate a practical methodology for radiative transfer simulations in disordered nanostructures. The results section of the paperis organized into six sections: (i) In the first section, we derive the formulas for polarization-and-orientation-averaged *C*_abs_, *C*_sca_, and μ_sca_ for arbitrary-shaped scatterers. The formulas are independent of the wave basis and, therefore, can be implemented by integral-equation methods for electromagnetic scattering, such as T-Matrix Method,^9^ BEM,^20^ or DDA.^21^ Based on these results, we evaluate the accuracy limits of other expressions for average light scattering commonly used in the literature.^22,23^ (ii) In the next section, we demonstrate that the formulas of average light scattering can be derived through the principles of fluctuational electrodynamics and, hence, can be computed through the mathematical methods used in studies of near-field thermal radiation,^24−27^ Casimir forces,^27^ and vacuum friction.^28,29^ In this context, we develop a computational application to numerically compute averaged light scattering,^30^ which is based on the fluctuating-surface-current BEM.^31,32^ (iii) In the following section, we validate the theory and simulation code for average scattering simulations against other analytical solutions.^33^ (iv) In the forth section, we analyze the advantages of the theory of average light scattering against brute-force averaging based on plane-wave simulations at different angles of incidence. (v) Next, we discuss how the three average light scattering parameters can be applied for modeling of radiative transfer in disordered nanostructures. (vi) The accuracy of the modeling framework is demonstrated in the final section, showing excellent agreement with optical measurements of polyethylene (PE) film composites with monoclinic vanadium dioxide [VO_2_(M)] microcrystals.

## Results

### Theory of Average Light Scattering of Randomly Oriented Particles

As discussed previously, light scattering from a collection of independent scatterers of arbitrary morphology and randomly oriented in space (Figure 1) is equivalent to the average light scattering over all orientations and light polarizations.^14^ We particularly focus on the average absorption cross section, ⟨*C*_abs_⟩, scattering cross section, ⟨*C*_sca_⟩, and asymmetry parameter, ⟨μ_sca_⟩, where , the *P* index runs over the two orthogonal polarizations, and **k̂**^i^ is the direction of the incident field. The asymmetry parameter, μ_sca_, defines the degree of anisotropy of scattering relative to **k̂**^i^:^11^
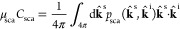
where **k̂**^s^ is the direction of the scattered field and *p*_sca_(**k̂**^s^, **k̂**^i^) is the scattering phase function.^14^ By definition of *p*_sca_, . Thus, μ_sca_ > 0 (μ_sca_ < 0) represents cases of forward (backward) anisotropic scattering and μ_sca_ = 0 represents isotropic scattering.

Our derivations are based on the Lippmann–Schwinger approach, a general formalism for electromagnetic scattering phenomena.^27^ In this approach, the scattered fields are given by  [in this notation, ], where **E**^i^ is the incident field,  is the free space Dyadic Green function, and  is the scattering operator (Supporting Information, eqs S4 and S5, respectively). The mathematical form of  is dictated by the expansion basis and the geometry and optical properties of the scatterer. For example, using the spherical wave basis, the  operator for a spherical particle is , where **f**_*l*_^reg^ is the spherical waves regular at the origin, *T*_ll_ is the Mie scattering coefficient, and † is the conjugate transpose operator.^27^ In integral-equation methods for electromagnetic scattering, such as the T-Matrix Method,^9^ BEM,^20^ or DDA,^21^ the form of  and  has to be computed prior to any scattering calculation.

In this context, the formulas of *C*_abs_ and *C*_sca_ for an incident plane wave of amplitude *E*_0_ are given by (details in Supporting Information, Section 1.1):^26,34^
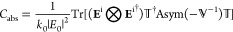
1a
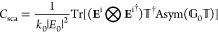
1bwhere *k*_0_ is the wavevector in free space, ⊗ is the tensor product, and  is the particle’s induced potential (Supporting Information, eq S2), with . The operators  and , where  is the adjoin of , represent the Hermitian and anti-Hermitian part of , respectively. The trace is defined as .

The formula of μ_sca_ is, to the best of our knowledge, presented here for the first time:
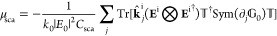
1cwhere the index *j* in **k̂**_*j*_^i^ and in the partial derivative ∂_*j*_, represents the global coordinates of the system (e.g., *j* = *x*, *y*, *z*). The formula is derived from the Lorentz force of the scattered fields over the induced currents in an object (Supporting Information, eq S10).

Because the operators  and  are independent of the direction of the incident field, ⟨*C*_abs_⟩ and ⟨*C*_sca_⟩ are uniquely determined by ⟨**E**^i^ ⊗ **E**^i^†^^⟩, also known as the free space self-correlator.^27^ For **E**^i^ in the form of monocromatic plane waves, this term is given by the following (see derivation in Supporting Information, eq S8):
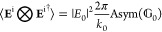
2

Similarly, ⟨μ_sca_⟩ requires the following (see derivation in Supporting Information, eq S11):
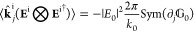
3

Using eqs 2 and 3, we derive the following expressions:
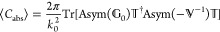
4a
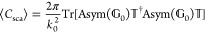
4b
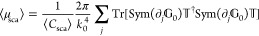
4cwhich represent a universal recipe for average light scattering, compatible with integral-equation methods for electromagnetic scattering. As an illustrative example, in the Supporting Information, we derive the respective formulas for BEM and T-Matrix using these expressions (Supporting Information, sections 1.2 and 1.3, respectively).

The relations, eqs 4a, 4b, and 4c, can be easily generalized for clustered particles and heterogeneous composites containing different types of particles (Supporting Information, section 1.4). The light scattering parameters for an individual particle particle *n* in the cluster, that is, ⟨*C*_abs_^*n*^⟩, ⟨*C*_sca_^*n*^⟩, and ⟨μ_sca_^*n*^⟩, are also obtained directly from these relations (details in Supporting Information, section 1.5).

We finalize this section by discussing a common approximation for ⟨*C*_abs_⟩ and ⟨*C*_sca_⟩ found in the literature:^22,23^
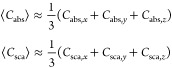
where *C*_abs,*j*_ and *C*_sca,*j*_ (*j* = *x*, *y*, *z*) are, respectively, the absorption and scattering cross sections for a plane wave polarized in the *j*-direction. As demonstrated in the Supporting Information (section 1.6), the expression ⟨*C*_abs_⟩ ≈ 1/3(*C*_abs,*x*_ + *C*_abs,*y*_ + *C*_abs,*z*_) corresponds to a particular case of eq 4a for subwavelength objects, while the analogous expression for ⟨*C*_sca_⟩ holds only if the polarizability of the object is a diagonal tensor.

### Average Light Scattering Derived from Fluctuational Electrodynamics

The trace formulas for ⟨*C*_abs_⟩, ⟨*C*_sca_⟩, and ⟨μ_sca_⟩ share many similarities with the relations found in studies of fluctuational electrodynamics, namely, thermal radiation and nonequilibrium electromagnetic forces.^27,31^ For example, in the framework of fluctuational electrodynamics, the thermal radiation absorbed by an isolated object, *P*_abs_^th^, is^27^
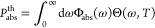
where  and , where *T* is the temperature of the environment, ω is the angular frequency, *k*_B_ is the Boltzmann constant, and ℏ is the reduced Planck constant. On the other hand, from light scattering theory,^11^*P*_abs_^th^ = ∫_0_^∞^ dω4π⟨*C*_abs_⟩*B*_ω_(*T*), where  is the Planck distribution. This leads to the following relation:
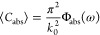
5This formula can be easily adapted to compute ⟨*C*_sca_⟩ by replacing  for .

Another relation comes from the electromagnetic friction induced by a moving photon gas on a stationary object.^28,29^ To a first order approximation, the friction coefficient γ_f_, can be expressed in terms of ⟨*C*_abs_⟩, ⟨*C*_sca_⟩, and ⟨μ_sca_⟩ as follows (Supporting Information, section 2.1):
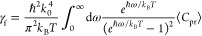
6where ⟨*C*_pr_⟩ = ⟨*C*_ext_⟩ – ⟨μ_sca_*C*_sca_⟩ and ⟨*C*_ext_⟩ = ⟨*C*_abs_⟩ + ⟨*C*_sca_⟩ are the average radiation pressure and extinction cross sections, respectively.^11^

As illustrated by eqs 5 and 6, the formulas of average scattering can be obtained through the principles of fluctuational electrodynamics. Consequently, the vast library of analytical solutions^27,28^ and numerical algorithms^31,35^ developed in the context of nonequilibrium energy and momentum transfer can be used to compute ⟨*C*_abs_⟩, ⟨*C*_sca_⟩, and ⟨μ_sca_⟩. For example, the thermal DDA^35^ and fluctuating current BEM^31^ for thermal radiation simulations, have explicit relations for Φ_abs_ that can be adapted to compute ⟨*C*_abs_⟩ and ⟨*C*_sca_⟩. On the other hand, the fluctuating current BEM also includes routines to compute ,^32^ which can be adapted for ⟨μ_sca_⟩.

In this context, we developed a computational code for average light scattering simulations, based on the fluctuating surface-current BEM formulation for nonequilibrium energy and momentum transfer.^31,32^ The code is implemented as an application of the SCUFF-EM software^36^ and can be acceded here.^30^ Similar to other simulation tools based on BEM,^31,32,37,38^ our code supports objects of arbitrary morphology and groups of objects (Supporting Information, Figure S1), offering a convenient platform to explore the full potential of the average scattering theory presented here.

### Validation of Average Light Scattering Theory against Analytical Solutions

**Figure 2:**
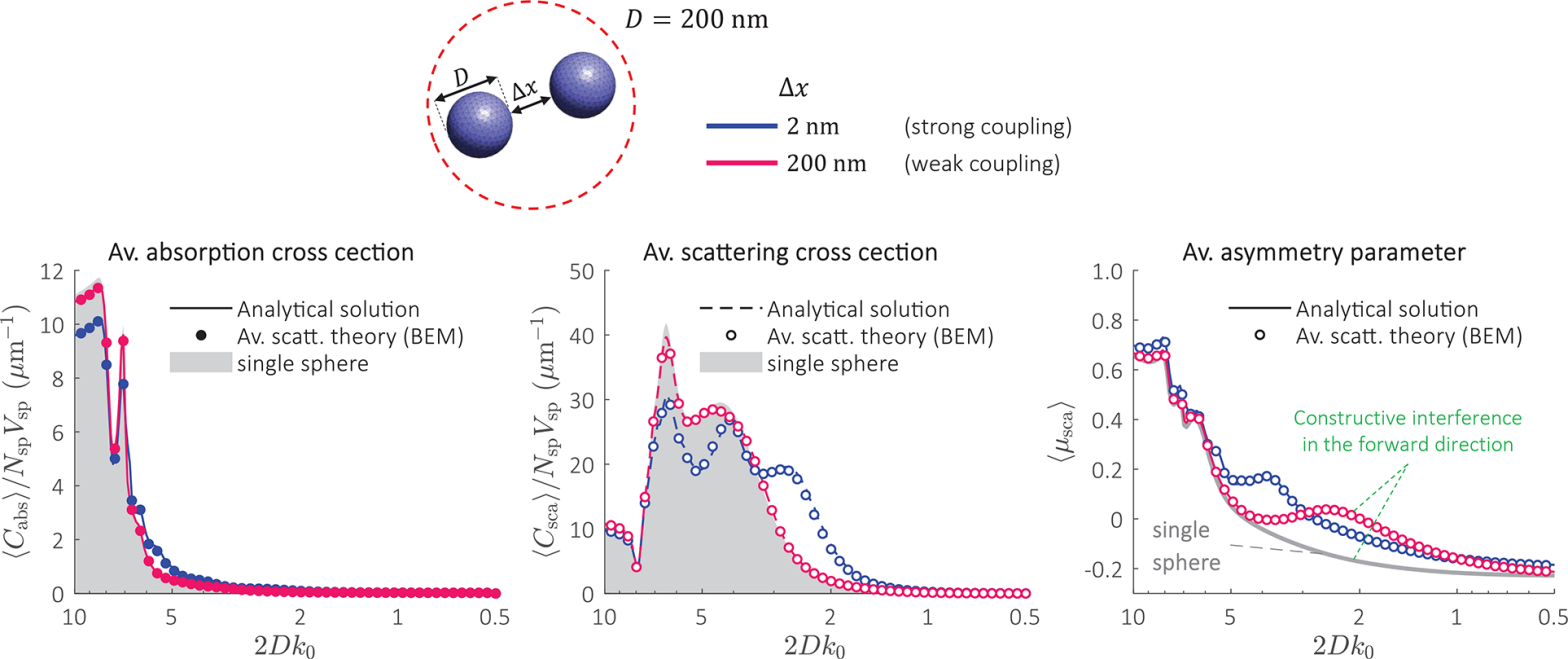
Average light scattering of randomly oriented silver sphere dimer. ⟨*C*_abs_⟩, ⟨*C*_sca_⟩, and ⟨μ_sca_⟩ of the dimer as a function of the size parameter 2D*k*_0_ (*k*_0_ = 2π/λ). The curves ⟨*C*_abs_⟩ and ⟨*C*_sca_⟩ are normalized to the number of spheres, *N*_sp_ = 2, and spheres volume, *V*_sp_, for direct comparison with the absorption and scattering cross section of a single sphere. The results are computed by the BEM code for average light scattering simulations^30^ and compared against the analytical solution.^33^ The scattering parameters of a single sphere, that is, absorption and scattering cross section normalized to the sphere’s volume (gray areas) and asymmetry parameter (gray curve) are computed from Mie-scattering^11^ and plotted as a reference. The dielectric constant of silver can be found elsewhere.^39^

To validate the average scattering theory and BEM simulation code, we consider the problem of light scattering by a randomly oriented sphere dimer (Figure 2), which has a known solution under the T-matrix approach.^33^ The dimer consists of two silver spheres of diameter, *D* = 200 nm, separated by a gap of (i) Δ*x* = 2 nm and (ii) Δ*x* = 200 nm. The light scattering parameters of a single sphere obtained from Mie Scattering Theory^11^ are also plotted as a reference.

The results from the average scattering theory show excellent agreement with the analytical solution by Mishchenko et al.^33^ (Figure 2). When Δ*x* = 2 nm, the effects of electromagnetic coupling dominate and the average scattering curves of the dimer largely deviate from the response of a single sphere. When Δ*x* = 200 nm, the coupling effects weaken and both ⟨*C*_abs_⟩ and ⟨*C*_sca_⟩ approach the response of a single sphere. However, this is not the case for ⟨μ_sca_⟩. Similar to the optical phenomenon observed in dilute particle media,^40^ the scattered fields interfere constructively in the forward direction, which explains the discrepancy between the ⟨μ_sca_⟩ curves.

As a second test, we consider the problems of average scattering from randomly oriented oblate and prolate spheroids, which has a known solution under the T-matrix approach.^33^ The results are displayed in the Supporting Information (Figure S3), showing excellent agreement up to *L*_max_/λ ≳ 2.5, where *L*_max_ is the longest ellipsoid axis. At shorter wavelengths, there is a discrepancy of ∼5% associated with the size of the mesh used in BEM simulations. Even with this discrepancy, the results are consistent with the predictions from the analytical solution, allowing to validate the theory presented here.

### Comparison against Brute-Force Averaging Methods

Consider, for example, the computation of ⟨*C*_sca_⟩ by averaging over many plane-wave simulations at different angles of incidence (brute-force averaging). In terms of the Lippmann–Schwinger approach to scattering and using eq 1b, this technique can be expressed as
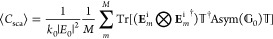
7where *m* indicates a plane-wave simulation at a particular angle of incidence and polarization and *M* is the total number simulations.

Similar to other formulas derived in this work, eq 7 is a general recipe that illustrates how to compute ⟨*C*_sca_⟩ using brute-force averaging. From this formula, we can deduct the steps required by integral-equation methods of electromagnetic scattering, that is:

1. Compute the expansion of **E**_*m*_^i^ using the particular basis.
2. Repeat “step 1” *M* times.
3. Determine the mathematical form of  and  for a particular expansion basis.
4. Replace **E**_*m*_^i^, , and  into eq 7.

On the other hand, as illustrated by eq 4b, the theory of average light scattering does not require **E**_*m*_^i^. Consequently, the computation of ⟨*C*_sca_⟩ is reduced to only two steps:

1. Determine the mathematical form of  and , for a particular expansion basis.
2. Replace  and  into eq 4b.

Because this approach requires less steps than brute-force averaging, it could potentially enable more efficient computation of ⟨*C*_sca_⟩. The same arguments applies for ⟨*C*_abs_⟩ and ⟨μ_sca_⟩. Note, however, that the computational cost of each technique is relative to the particular algorithm implemented. Thus, specially for problems where *M* is small, brute-force averaging could be performed with higher computational speed and less memory requirements.

### Radiative Transfer Modeling for Random Media with Scatterers of Arbitrary Morphology

For unpolarized light and under the independent scattering approximation, the steady-state RTE for randomly oriented scatterers in a nonabsorbing host is^9^
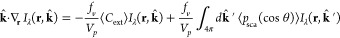
8where *I*_λ_ is the specific radiative intensity (defined as the energy flux per unit solid angle), *f*_*v*_ is the volume fraction, *V*_*p*_ is the effective volume of the scatterers, **k̂**·∇_**r**_*I*_λ_(**r**, **k̂**) is the rate of change of *I*_λ_ at the position **r** and direction **k̂**; and ⟨*p*_sca_(cos θ)⟩ is the orientation and polarization averaged scattering phase function, where cos θ = **k̂**·**k̂**′.

Commonly, solutions of eq 8 consider approximated expressions for the phase function in terms of μ_sca_.^12^ For example, the Henyey–Greenstein model,^12^
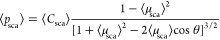
9is widely used in simulations methods, such as Monte Carlo,^7^ adding-doubling,^41^ and discrete ordinate.^12^

As evidenced by eqs 8 and 9, the RTE and the average light scattering parameters ⟨*C*_abs_⟩, ⟨*C*_sca_⟩, and ⟨μ_sca_⟩ constitute a complete set to model radiative transfer in composites with scatterers of arbitrary morphology. As demonstrated in the next section, this modeling framework enables the quantitative prediction of the macroscopic radiative properties of a composite, such as the total transmittance (*T*_tot_), specular transmittance (*T*_spec_), and total reflectance (*R*_tot_).

### Validation of Radiative Transport Simulation against Experiments

**Figure 3:**
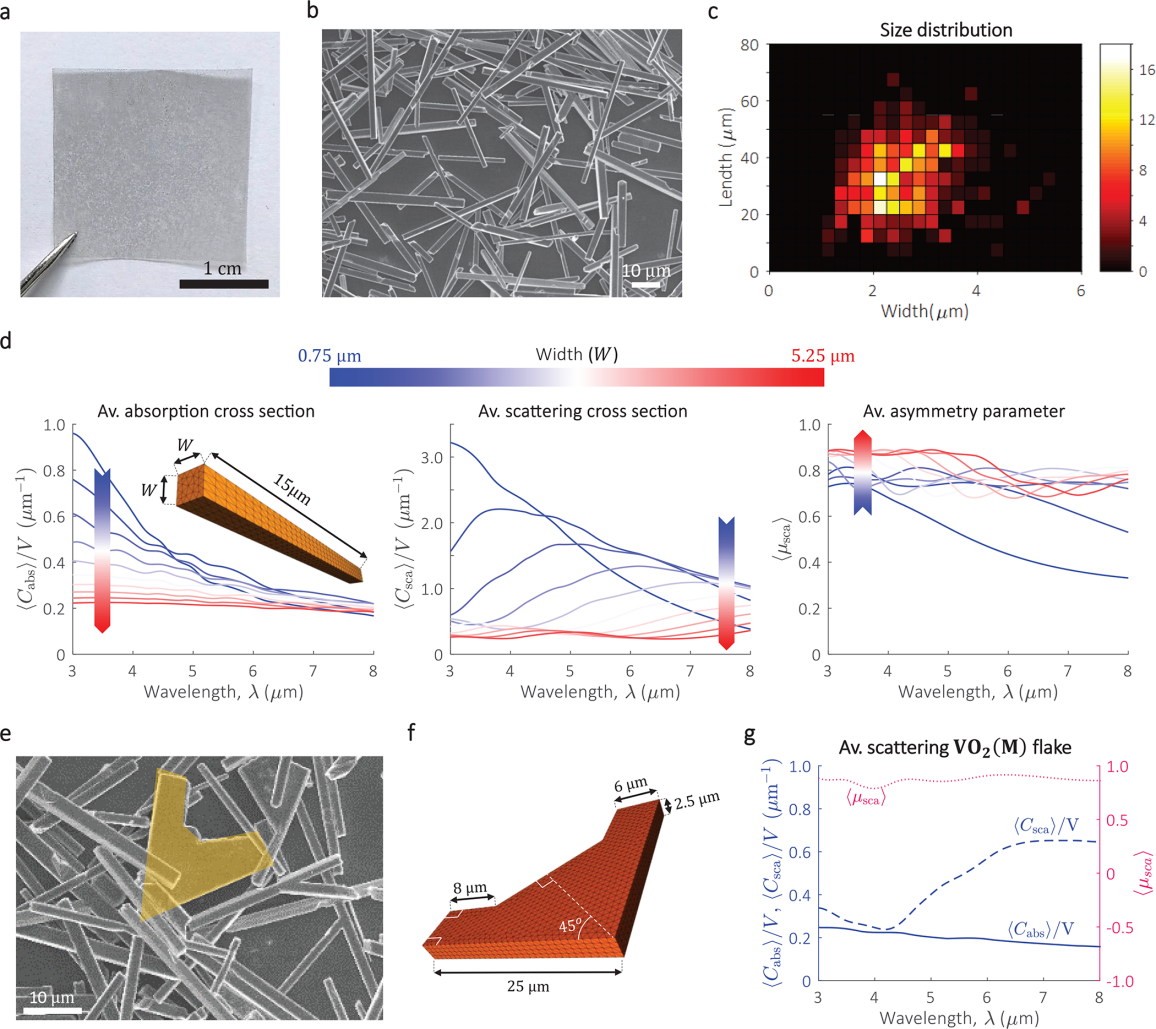
Characterization and average scattering simulations of monoclinic vanadium dioxide [VO_2_(M)] microcrystals embedded into a polyethylene (PE) matrix. (a) Photograph of VO_2_(M)/PE composite film. (b) SEM of as-grown VO_2_(M) crystals, which is mainly composed of VO_2_(M) bars. (c) Size distribution of VO_2_ bars. (d) ⟨*C*_abs_⟩, ⟨*C*_sca_⟩, and ⟨μ_sca_⟩ of VO_2_(M) bars of fixed length, *L* = 15 μm, and variable width, *W* = 0.75–5.25 μm in steps of 0.5 μm. The refractive index of the host, *n*_host_ = 1.5. The average scattering parameters showed similar dependence to *W* for other values of *L* (not shown here). The legend is given by the color bar at the top of the curves. (e) An example of one of the VO_2_(M) crystals with flake morphology is found in the characteristic sample. (f) Computational representation of the VO_2_(M) flakes, which was considered for the average scattering simulations. (g) Simulated average light scattering of the VO_2_(M) flake. For all average scattering simulations, the refractive index of the host, *n*_host_ = 1.5, and the refractive index of the VO_2_(M) bars was obtained from the literature (see “film2” in Wan et al.^42^).

We demonstrate the accuracy of the previously discussed modeling framework by comparing the radiative transfer simulations against optical measurements of a composite based on VO_2_(M) microcrystals embedded in a polyethylene (PE) matrix (Figure 3a). We considered VO_2_(M) microcrystals given its well-defined and highly anisotropic morphology (Figure 3b), providing an ideal scenario to validate the theory of average scattering and radiative transfer modeling. Additionally, the refractive index^42,43^ and size of microcrystals, ensures a significant contribution from both absorption and scattering in the mid infrared (IR) spectrum.^11^

First, we derived the average light scattering parameters of the VO_2_(M) microcrystals ensemble using a characteristic sample (Supporting Information, Figure S4c). The size distribution of the bars length (*L*) and width (*W*) is shown in Figure 3c, which assumes bars of square cross section. We calculated ⟨*C*_abs_⟩, ⟨*C*_sca_⟩, and ⟨μ_sca_⟩ of single VO_2_(M) bars for λ = 3–8 μm, considering the range of *W* and *L* dictated by the size distribution. The spectrum λ > 8 μm is excluded in the simulations due to the large uncertainty in the refractive index of VO_2_(M), which is strongly conditioned by crystal orientation, growth method, strain, and partial oxidation.^42^ As shown in Figure 3d, ⟨*C*_abs_⟩, ⟨*C*_sca_⟩, and ⟨μ_sca_⟩ are strongly sensitive to *W*. On the other hand, the three parameters are less sensitive to changes in *L*, with negligible variations for *L* > 15 μm (Supporting Information, Figure S5). In addition to the VO_2_(M) bars, we noted small traces of VO_2_(M) flakes in the sample, such as the one shown in Figure 3e. These VO_2_(M) flakes are represented by the computational model in Figure 3f, with the simulated average scattering parameters shown in Figure 3g.

**Figure 4:**
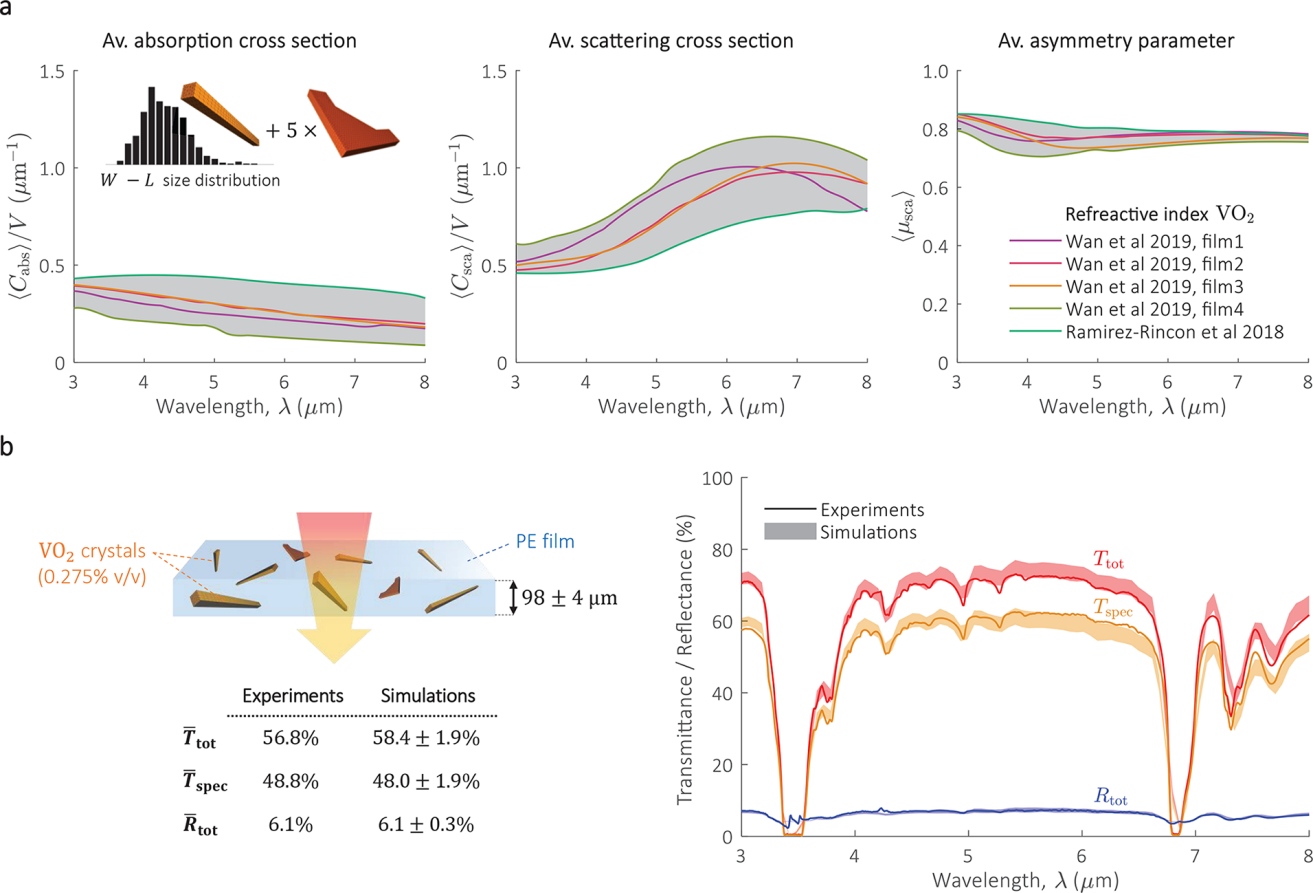
Radiative transfer modeling of VO_2_(M)/PE film composite. (a) ⟨*C*_abs_⟩, ⟨*C*_sca_⟩, and ⟨μ_sca_⟩ of VO_2_(M) microcrystal ensemble calculated for five different refractive indexes of VO_2_(M), as reported by Wan et al. 2019^42^ (labeled as “film1”, “film2”, “film3”, and “film4”), and Ramirez-Rincon et al. 2018.^43^ The gray areas mark the upper and lower limit due to variations in the refractive index. For a given refractive index, the curves ⟨*C*_abs_⟩, ⟨*C*_sca_⟩, and ⟨μ_sca_⟩ were obtained as indicated by the schematic (left figure, inset), that is, average scattering simulations of bars weighted by the size distribution + average scattering of five flakes. Further details in the Supporting Information, section 4.3. The curves ⟨*C*_abs_⟩ and ⟨*C*_sca_⟩ are normalized to the volume of the ensemble *V* (Supporting Information, eq S31). (b) Validation of radiative transfer theory, showing *T*_tot_, *T*_spec_, and *R*_tot_ of a VO_2_(M)/PE composite film, as obtained from optical measurements (solid lines) and simulations (filled areas). The optical properties of the PE film were extracted from optical measurements on a clear film (see Supporting Information, section 4.4). For the simulations, the absorption of PE is considered through the extinction coefficient κ_host_ (see Methods). The composite is based on a 98 ± 4 μm thick PE film with 0.275% v/v of VO_2_(M) microcrystals. The upper (lower) limit in the filled areas are the results of variations in the refractive index of VO_2_(M) and thickness of the film (98 – 4 or 98 + 4 μm).

The parameters ⟨*C*_abs_⟩, ⟨*C*_sca_⟩, and ⟨μ_sca_⟩ of the VO_2_(M) microcrystals ensemble (Figure 4a), were estimated using the average scattering simulations of individual bars and the flake, together with the size distribution. We repeated this procedure for five different refractive indexes reported in the literature^42,43^ in order to consider the variations in the optical properties of VO_2_(M). Using the parameters ⟨*C*_abs_⟩, ⟨*C*_sca_⟩, and ⟨μ_sca_⟩ of the VO_2_(M) microcrystals ensemble, together with Monte Carlo simulations of radiative transfer (see details in Methods), we estimate *T*_tot_, *T*_spec_, and *R*_tot_ of a VO_2_(M)/PE composite film (Figure 4b). The results are shown by filled areas, representing the upper and lower limits associated with the variations of the refractive index of VO_2_(M) and thickness of the film. The optical measurements show excellent agreement with the range predicted by simulations, which is further confirmed by comparing the spectral mean of *T*_tot_, *T*_spec_, and *R*_tot_ (table in Figure 4b). The accuracy of the simulation is further confirmed through a second test, which considers a composite film with a double concentration of VO_2_(M) microcrystals (Supporting Information, Figure S7).

## Conclusion

We presented a universal theory to predict the average light scattering from randomly oriented objects with arbitrary shape. The formulas of ⟨*C*_abs_⟩, ⟨*C*_sca_⟩, and ⟨μ_sca_⟩ can be implemented by any integral-equation method for electromagnetic scattering. Moreover, because these relations are exclusively defined in terms of the operators  and , they could lead to a more efficient computation of average scattering than brute-force techniques. The general form of the average scattering formulas also provides a convenient landscape to explore the fundamental limits of scattering in random systems. For example, in analogy to the studies of scattering and absorption bounds,^26,34^ the limits of forward or backward scattering of randomly oriented particles can be explored through the asymmetry parameter formula (eq 4c).

The demonstrated connection between average light scattering and fluctuational electrodynamics enables to extend the theory to other parameters of interest. For example, a formula for the average scattering of moving objects can be extracted from the theory of electromagnetic friction in objects at relative motion.^28^ Alternatively, other expressions can be extracted directly through the self-correlators in eqs 2 and 3, in a similar fashion than the relations obtained from the fluctuation–dissipation theorem.^25−27^

The parameters ⟨*C*_abs_⟩, ⟨*C*_sca_⟩, and ⟨μ_sca_⟩ are also practical for radiative transfer simulations for unpolarized light, enabling an accurate prediction of the optical properties of composites with scatterers of arbitrary shape, as demonstrated in the study of VO_2_(M)/PE composite films. The radiative transfer formula for randomly oriented particles (eq 8) can be extended to consider other effects present in the light transport process. For example, the emitted thermal radiation from scatterers can be represented through the term  at the right-hand side of the equation.^12^ Similarly, the absorption of the host can be included through the term −2*k*_0_κ_host_*I*_λ_(**r**, **k̂**) at the right-hand side of eq 8.

The methodology used in the study of VO_2_(M)/PE composite films can be also applied to other composite media, with either dielectrics^7^ or metal scatterers,^10^ providing that the distance between particles is large enough to ignore the effects of short-range correlations. For more complex problems, such as clustered particles or more dense particle distributions,^44^ the methodology can be extended using the formulation for multiple objects (Supporting Information, section 1.4). In this case, ⟨*C*_abs_⟩, ⟨*C*_sca_⟩, and ⟨μ_sca_⟩ must be obtained from simulations over a properly chosen collection of particles that better represent the effects from short-range correlations. The method, thus, could provide key insights to many problems in disordered nanophotonics, such as the effects of agglomeration into the optical absorption of gold nanostars or the impact of multiple scattering in the light trapping of heterostructured photocatalysts, as we will discuss in future works.

## Methods

### Fabrication and Characterization of VO_2_(M)/PE Composite Films

The composite was fabricated by dry mixing of VO_2_(M) microcrystals with low-density PE (LDPE; 42607, Sigma-Aldrich) and ultrahigh-molecular-weight PE (UHMWPE; 429015, Sigma-Aldrich) at a weight ratio of VO_2_(M)/LDPE/UHMWPE = 1:40:40. The mixture was then melt-pressed into a film at 200 °C. The VO_2_(M) crystals were produced by hydrothermal synthesis using our previously developed procedure.^45^ The phase of the crystals was confirmed by X-ray diffraction and Raman spectroscopy (Supporting Information, Figure S4a and b, respectively). The volume fraction of the VO_2_(M) microcrystals was estimated from the weight ratio and the densities of VO_2_(M) (4.230 g/cm^3^),^46^ LDPE (0.925 g/cm^3^), and UHMWPE (0.940 g/cm^3^). A micrometer was used to characterize the thickness of the film. The reported thickness corresponds to five measurements on different sections of the sample.

### Optical Measurements

The total and specular transmittance and total reflectance of the VO_2_(M)/polyethylene composite film were measured with a Fourier transform infrared spectrometer (IRTracer-100, Shimadzu) and a mid-IR integrating sphere (Pike Technologies).

### BEM Average Scattering Simulations

Simulations of orientation- and polarization-average light scattering were performed using the SCUFF-EM application AVESCATTER.^30^ SCUFF-EM^36^ is an open-source software for electromagnetic simulations based on the BEM. The meshing of the objects is based on triangular panels and was carried by GMSH.^47^

### Monte Carlo Simulations of Radiative Transfer

Radiative transfer simulations were performed by our own code for Monte Carlo simulations of unpolarized light. The algorithm consists of simulating the trajectories of many individual photons as they interact with particles and interfaces until they are either absorbed by particles or exit the simulation domain. The initial condition of each photon is given by the position and direction of the light source. At each simulation step, the optical path (Λ_photon_) and fate of a photon are estimated by selecting the shortest path between the particle’s scattering (Λ_sca_) and absorption (Λ_abs_), the absorption of the host (Λ_host_), or diffraction (Λ_Fresnel_), where
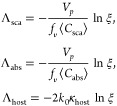
and ξ is a random number between 0 and 1; Λ_Fresnel_ is given by the shortest distance between the photon and an interface. In materials with more than one kind of particle, Λ_abs_ = min(Λ_abs_^*i*^) and Λ_sca_ = min(Λ_sca_^*n*^), where Λ_abs_^*n*^ and Λ_sca_^*n*^ are, respectively, the absorption and scattering path from the particle *n*.

In the case of diffraction (Λ_photon_ = Λ_Fresnel_), a photon is either reflected or transmitted by a random selection, with the probabilities of each event proportional to the respective energy flux defined by Fresnel laws. If the photon is absorbed by a particle (Λ_photon_ = Λ_abs_) or the host material (Λ_photon_ = Λ_host_), the event is terminated and the simulation continues with a new photon at the initial conditions. For a scattered photon (Λ_photon_ = Λ_sca_), the new direction is determined by^7^
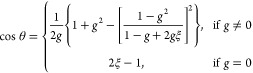
where *g* = ⟨μ_sca_⟩.

In all our simulations, we considered a slab with a large surface area in order to represent a 2D problem. As a criteria, we selected the smallest surface area by which no photon escapes through the edges. Two large monitors, above and below the slab, measure the total reflectance and transmittance, respectively. The specular transmittance was measured with a third small monitor (1 nm × 1 nm) at a 1 mm distance below the slab. In all the simulations, we considered 1000000 photons per wavelength. For the validation of our code, refer to Supporting Information, section 5.

## Supporting Information Available

Details of the derivation of average light scattering formulas for single objects, multiple objects, approximation for small objects, surface-current BEM and T-Matrix; Simulation of average light scattering for randomly oriented gold nanostars (single and clustered) as a demonstration of the BEM simulation code; Derivation of the vacuum friction formula; Validation of the theory against the analytical solution for average light scattering of randomly oriented spheroids; Details associated with the study of light transport in the VO_2_(M)/PE composite, including XRD, Raman shift, and SEM of as-grown VO_2_(M) microcrystals, average light scattering of VO_2_(M) bars as a function of *L*, further details regarding the calculation of average light scattering of the microcrystal ensemble, estimation of the refractive index and scattering properties of PE films, and radiative properties of a second VO_2_(M)/PE film with higher concentration of VO_2_(M) particles; Validation of Monte Carlo code for radiative transfer simulations (PDF)

## References

[ref1] Langer J.; et al. Present and future of surface-enhanced Raman scattering. ACS Nano 2020, 14, 28–117. 10.1021/acsnano.9b04224. 31478375 PMC6990571

[ref2] Cheng L.; Wang C.; Feng L.; Yang K.; Liu Z. Functional nanomaterials for phototherapies of cancer. Chem. Rev. 2014, 114, 10869–10939. 10.1021/cr400532z. 25260098

[ref3] Low J.; Yu J.; Jaroniec M.; Wageh S.; Al-Ghamdi A. A. Heterojunction Photocatalysts. Adv. Mater. 2017, 29, 1601694 10.1002/adma.201601694. 28220969

[ref4] Zhao D.; Aili A.; Zhai Y.; Xu S.; Tan G.; Yin X.; Yang R. Radiative sky cooling: Fundamental principles, materials, and applications. Appl. Phys. Rev. 2019, 6, 021306 10.1063/1.5087281.

[ref5] Tao P.; Ni G.; Song C.; Shang W.; Wu J.; Zhu J.; Chen G.; Deng T. Solar-driven interfacial evaporation. Nat. Energy 2018, 3, 1031–1041. 10.1038/s41560-018-0260-7.

[ref6] Cai W.; Shalaev V. Optical Metamaterials: Fundamentals and Applications; Springer: New York, USA, 2009; p 200.

[ref7] Wang L.; Jacques S. L.; Zheng L. MCML—Monte Carlo modeling of light transport in multi-layered tissues. Comput. Methods Programs Biomed. 1995, 47, 131–146. 10.1016/0169-2607(95)01640-F. 7587160

[ref8] Ishimaru A. Wave Propagation and Scattering in Random Media, 1st ed.; Oxford University Press: Oxford, U.K., 1997; p 600.

[ref9] Tsang L.; Kong J. A.; Ding K.-H.; Ao C. O. Scattering of Electromagnetic Waves: Theories and Applications; John Wiley & Sons, Inc.: New York, U.S.A., 2000; p 445.

[ref10] Hogan N. J.; Urban A. S.; Ayala-Orozco C.; Pimpinelli A.; Nordlander P.; Halas N. J. Nanoparticles heat through light localization. Nano Lett. 2014, 14, 4640–4645. 10.1021/nl5016975. 24960442

[ref11] Bohren C. F.; Huffman D. R. Absorption and Scattering of Light by Small Particles; John Wiley & Sons, Inc., 1998; p 544.

[ref12] Stamnes K.; Thomas G. E.; Stamnes J. J. Radiative Transfer in the Atmosphere and Ocean, 2nd ed.; Cambridge University Press: Cambridge, U.K., 2017; p 531.

[ref13] Tsang L.; Kong J. A.; Ding K.-H.; Ao C. O. Scattering of Electromagnetic Waves: Numerical Simulations; John Wiley & Sons, Inc., 2001; p 736.

[ref14] Hulst H. C. Light Scattering by Small Particles; Dover Publications, Inc.: New York, U.S.A., 1981; p 470.

[ref15] Suryadharma R. N.; Rockstuhl C. Predicting observable quantities of self-assembled metamaterials from the T-Matrix of its constituting meta-atom. Mater. 2018, 11, 213 10.3390/ma11020213. PMC5848910 29385711

[ref16] Bi L.; Yang P.; Kattawar G. W.; Mishchenko M. I. A numerical combination of extended boundary condition method and invariant imbedding method applied to light scattering by large spheroids and cylinders. J. Quant. Spectrosc. Radiat. Transfer 2013, 123, 17–22. 10.1016/j.jqsrt.2012.11.033.

[ref17] Mackowski D. W.; Mishchenko M. I. A multiple sphere T-matrix Fortran code for use on parallel computer clusters. J. Quant. Spectrosc. Radiat. Transfer 2011, 112, 2182–2192. 10.1016/j.jqsrt.2011.02.019.

[ref18] Khlebtsov N. G. Orientational averaging of light-scattering observables in the T-matrix approach. Appl. Opt. 1992, 31, 5359 10.1364/AO.31.005359. 20733719

[ref19] Fazel-Najafabadi A.; Schuster S.; Auguié B. Orientation averaging of optical chirality near nanoparticles and aggregates. Phys. Rev. B 2021, 103, 115405 10.1103/PhysRevB.103.115405.

[ref20] Reid M. T.; Johnson S. G. Efficient Computation of Power, Force, and Torque in BEM Scattering Calculations. IEEE Trans. Antennas Propag. 2015, 63, 3588–3598. 10.1109/TAP.2015.2438393.

[ref21] Yurkin M. A.; Hoekstra A. G. The discrete dipole approximation: An overview and recent developments. J. Quant. Spectrosc. Radiat. Transfer 2007, 106, 558–589. 10.1016/j.jqsrt.2007.01.034.

[ref22] Santiago E. Y.; Besteiro L. V.; Kong X.-T.; Correa-Duarte M. A.; Wang Z.; Govorov A. O. Efficiency of Hot-Electron Generation in Plasmonic Nanocrystals with Complex Shapes: Surface-Induced Scattering, Hot Spots, and Interband Transitions. ACS Photonics 2020, 7, 2807–2824. 10.1021/acsphotonics.0c01065.

[ref23] Muskens O. L.; Bachelier G.; Del Fatti N.; Vallée F.; Brioude A.; Jiang X.; Pileni M. P. Quantitative absorption spectroscopy of a single gold nanorod. J. Phys. Chem. C 2008, 112, 8917–8921. 10.1021/jp8012865.

[ref24] Liu B.; Li J.; Shen S. Resonant Thermal Infrared Emitters in Near- and Far-Fields. ACS Photonics 2017, 4, 1552–1557. 10.1021/acsphotonics.7b00336.

[ref25] Cuevas J. C.; García-Vidal F. J. Radiative Heat Transfer. ACS Photonics 2018, 5, 3896–3915. 10.1021/acsphotonics.8b01031.

[ref26] Molesky S.; Jin W.; Venkataram P. S.; Rodriguez A. W. T Operator Bounds on Angle-Integrated Absorption and Thermal Radiation for Arbitrary Objects. Phys. Rev. Lett. 2019, 123, 257401 10.1103/PhysRevLett.123.257401. 31922767

[ref27] Krüger M.; Bimonte G.; Emig T.; Kardar M. Trace formulas for nonequilibrium Casimir interactions, heat radiation, and heat transfer for arbitrary objects. Phys. Rev. B 2012, 86, 115423 10.1103/PhysRevB.86.115423.

[ref28] Golyk V. A.; Krüger M.; Kardar M. Linear response relations in fluctuational electrodynamics. Phys. Rev. B 2013, 88, 155117 10.1103/PhysRevB.88.155117.

[ref29] Mkrtchian V.; Parsegian V. A.; Podgornik R.; Saslow W. M. Universal thermal radiation drag on neutral objects. Phys. Rev. Lett. 2003, 91, 220801 10.1103/PhysRevLett.91.220801. 14683225

[ref30] Ramirez-Cuevas F. V. AVESCATTER: A SCUFF-EM application for light scatttering of randomly oriented particles of arbitrary shape. https://github.com/PanxoPanza/scattering_random_orientation (accessed on 2022–01–25).

[ref31] Rodriguez A. W.; Reid M. T.; Johnson S. G. Fluctuating-surface-current formulation of radiative heat transfer: Theory and applications. Phys. Rev. B 2013, 88, 054305 10.1103/PhysRevB.88.054305.

[ref32] Reid M. T. H.; Miller O. D.; Polimeridis A. G.; Rodriguez A. W.; Tomlinson E. M.; Johnson S. G. Photon Torpedoes and Rytov Pinwheels: Integral-Equation Modeling of Non-Equilibrium Fluctuation-Induced Forces and Torques on Nanoparticles. arXiv 1708.01985 [physics.optics] 2017, na.

[ref33] Mishchenko M.; Travis L.; Mackowski D. T-Matrix Codes for Computing Electromagnetic Scattering by Nonspherical and Aggregated Particles. https://www.giss.nasa.gov/staff/mmishchenko/t_matrix.html (accessed on 2021–07–17).

[ref34] Molesky S.; Chao P.; Jin W.; Rodriguez A. W. Global T operator bounds on electromagnetic scattering: Upper bounds on far-field cross sections. Phys. Rev. Res. 2020, 2, 033172 10.1103/PhysRevResearch.2.033172.

[ref35] Edalatpour S.; Francoeur M. The Thermal Discrete Dipole Approximation (T-DDA) for near-field radiative heat transfer simulations in three-dimensional arbitrary geometries. J. Quant. Spectrosc. Radiat. Transfer 2014, 133, 364–373. 10.1016/j.jqsrt.2013.08.021.

[ref36] Reid M. T. H. SCUFF-EM: Free, open-source software for boundary-element analysis of problems in computational physics and engineering. https://homerreid.github.io/scuff-em-documentation/ (accessed on 2022–01–25).

[ref37] Solís D. M.; Taboada J. M.; Obelleiro F.; Liz-Marzán L. M.; García de Abajo F. J. Toward Ultimate Nanoplasmonics Modeling. ACS Nano 2014, 8, 7559–7570. 10.1021/nn5037703. 25077678

[ref38] Solís D. M.; Taboada J. M.; Obelleiro F.; Liz-Marzán L. M.; García De Abajo F. J. Optimization of Nanoparticle-Based SERS Substrates through Large-Scale Realistic Simulations. ACS Photonics 2017, 4, 329–337. 10.1021/acsphotonics.6b00786. 28239616 PMC5319398

[ref39] Babar S.; Weaver J. H. Optical constants of Cu, Ag, and Au revisited. Appl. Opt. 2015, 54, 477–481. 10.1364/AO.54.000477.

[ref40] Hecht E. Optics, 5th ed.; Pearson Education: Essex, U.K., 2016; p 714.

[ref41] Prahl S. A.; van Gemert M. J. C.; Welch A. J. Determining the optical properties of turbid media by using the adding–doubling method. Appl. Opt. 1993, 32, 559–568. 10.1364/AO.32.000559. 20802725

[ref42] Wan C.; et al. On the Optical Properties of Thin-Film Vanadium Dioxide from the Visible to the Far Infrared. Ann. Phys. 2019, 531, 1900188 10.1002/andp.201900188.

[ref43] Ramirez-Rincon J. A.; Gomez-Heredia C. L.; Corvisier A.; Ordonez-Miranda J.; Girardeau T.; Paumier F.; Champeaux C.; Dumas-Bouchiat F.; Ezzahri Y.; Joulain K.; Ares O.; Alvarado-Gil J. J. Thermal hysteresis measurement of the VO_2_ dielectric function for its metal-insulator transition by visible-IR ellipsometry. J. Appl. Phys. 2018, 124, 195102 10.1063/1.5049747.

[ref44] Hwang V.; Stephenson A. B.; Barkley S.; Brandt S.; Xiao M.; Aizenberg J.; Manoharan V. N. Designing angle-independent structural colors using Monte Carlo simulations of multiple scattering. Proc. Natl. Acad. Sci. U.S.A. 2021, 118, e2015551118 10.1073/pnas.2015551118. 33472972 PMC7848739

[ref45] Gurunatha K. L.; Sathasivam S.; Li J.; Portnoi M.; Parkin I. P.; Papakonstantinou I. Combined Effect of Temperature Induced Strain and Oxygen Vacancy on Metal-Insulator Transition of VO2 Colloidal Particles. Adv. Funct. Mater. 2020, 30, 2005311 10.1002/adfm.202005311.

[ref46] Jain A.; Ong S. P.; Hautier G.; Chen W.; Richards W. D.; Dacek S.; Cholia S.; Gunter D.; Skinner D.; Ceder G.; Persson K. A. Commentary: The materials project: A materials genome approach to accelerating materials innovation. APL Mater. 2013, 1, 011002 10.1063/1.4812323.

[ref47] Geuzaine C.; Remacle J. F. Gmsh: A 3-D finite element mesh generator with built-in pre- and post-processing facilities. Int. J. Numer. Methods Eng. 2009, 79, 1309–1331. 10.1002/nme.2579.

