# Supplementary material for: Universal Theory of Light Scattering of Randomly Oriented Particles: A Fluctuational-Electrodynamics Approach for Light Transport Modeling in Disordered Nanostructures
Source: ACS Photonics. 2022 Feb 4;9(2):672–81. doi: 10.1021/acsphotonics.1c01710 (PMC9097575; doi:10.1021/acsphotonics.1c01710)
Supplement: Supplementary file 1 — ph1c01710_si_001.pdf [file ph1c01710_si_001.pdf]

Supporting Information for "Universal Theory of Light Scattering of Randomly Oriented Particles: A Fluctuational-Electrodynamics Approach for Light Transport Modeling in Disordered Nanostructures"

Francisco V. Ramirez-Cuevas,[†,‡] Kargal L. Gurunatha,[†] Ivan P. Parkin,[¶] and Ioannnis Papakonstantinou[*,†]

†*Photonic Innovations Lab, Department of Electronic and Electrical Engineering, University College London, London, WC1E 7JE, United Kingdom*

‡*Center for Energy Transition, Facultad de Ingeniería y Ciencias, Universidad Adolfo Ibáñez, Santiago, 7941169, Chile*

¶*Department of Chemistry, University College London, London, WC1H 0AJ, United Kingdom*

E-mail: i.papakosntantinou@ucl.co.uk

Contents

1 Theory of average light scattering from random oriented particles S2

1.1 Formulation for a single object S2

1.2 Surface-current BEM formulation S6

1.3 Average light scattering in Spherical wave basis: T-matrix formulation . . . S9

1.4 Formulation for multiple objects S11
1.5 Formulation for an individual object in a cluster S12
1.6 Approximation for subwavelength particles S13

2 Average light scattering from fluctuational electrodynamics S15
2.1 Vacuum friction and averaged light scattering S15

3 Validation of average light scattering theory against analytical solutions S16
3.1 Average light scattering of randomly oriented spheroids S16

4 Validation of radiative transfer simulation against experiments S17
4.1 Characterization of as-grown VO_2(M) microcrystals S17
4.2 Average light scattering of VO_2(M) bars as a function of L. S18
4.3 Estimation of average light scattering of VO_2(M) microcrystal ensemble . . . S18
4.4 Prediction of refractive index and scattering from PE films S19
4.5 Radiative properties of VO_2(M)/PE composite films (Experiments and Simulations S21

5 Validation of Monte-Carlo Code S21

References S23

1 Theory of average light scattering from random oriented particles

1.1 Formulation for a single object

In the context of Lippmann-Schwinger approach,[1] the presence of a object in an infinitely extended medium 0 induces a perturbation to the incident (source) electric fields, $\mathbf{E}^{\mathrm{i}}$, that results in a scattered (induced) electric field, $\mathbf{E}^{\mathrm{s}}$, in medium 0. For simplicity, we consider

medium 0 as vacuum, with electrical permittivity, ε_0, and magnetic permeability, μ_0, while the object is characterized by linear electromagnetic properties, i.e., dielectric constant, $\mathbb{\epsilon}$, and relative permeability $\mathbb{\mu}$, which can be, in general, nonlocal complex tensors. The total electric field, $\mathbf{E}^{\mathrm{t}}$, that results from the superposition of $\mathbf{E}^{\mathrm{i}}$ and $\mathbf{E}^{\mathrm{s}}$, satisfies the following equation:[1]

$$\left[\mathbb{H}_0 - k_0^2\mathbb{I} - \mathbb{V}\right]\mathbf{E}^{\mathrm{t}} = 0, \tag{S1}$$

where $k_0 = \omega\sqrt{\varepsilon_0\mu_0}$ is the wavevector in free space, $\mathbb{I}$ is the identity operator, $\mathbb{H}_0 = \nabla\times\nabla\times$, and

$$\mathbb{V} = k_0^2\left(\mathbb{\epsilon} - \mathbb{I}\right) + \nabla\times\left(\mathbb{I} - \mathbb{\mu}^{-1}\right)\nabla\times \tag{S2}$$

is the induced potential by the object, with $\mathbb{V} = 0$ anywhere outside the object.

Solution of Eq. (S1) is given by:

$$\mathbf{E}^{\mathrm{t}} = \left(\mathbb{I} + \mathbb{G}_0\mathbb{T}\right)\mathbf{E}^{\mathrm{i}}, \tag{S3}$$

where $\mathbb{G}_0$ is the free space dyadic green function:[2]

$$\mathbb{G}_0\left(\mathbf{r}, \mathbf{r}'\right) = \left[\mathbb{I} + \frac{1}{k_0^2}\nabla\nabla\right]\frac{e^{i\mathbf{k}_0|\mathbf{r}-\mathbf{r}'|}}{4\pi|\mathbf{r}-\mathbf{r}'|}, \tag{S4}$$

which satisfies $\left[\mathbb{H}_0 - \mathbb{I}\right]\mathbb{G}_0 = \delta(r - r')$, and $\mathbb{T}$ is the scattering operator defined by:[1,3]

$$\mathbb{T} = \left[\mathbb{V}^{-1} - \mathbb{G}_0\right]^{-1} \tag{S5}$$

$\mathbb{T} = 0$ anywhere outside of the object n.

We begin our discussion with the definition of the absorption, P_{abs}, and scattering, P_{sca},

energy fluxes in terms of the $\mathbb{T}$-operator:[4]

$$P_{\text{abs}} = \frac{1}{2k_0 Z_0}\text{Tr}\left[\left(\mathbf{E}^{\text{i}} \otimes \mathbf{E}^{\text{i}\dagger}\right) \mathbb{T}^{\dagger} \text{ Asym}\left(-\mathbb{V}^{-1}\right) \mathbb{T}\right] \tag{S6a}$$

$$P_{\text{sca}} = \frac{1}{2k_0 Z_0}\text{Tr}\left[\left(\mathbf{E}^{\text{i}} \otimes \mathbf{E}^{\text{i}\dagger}\right) \mathbb{T}^{\dagger} \text{ Asym}\left(\mathbb{G}_0\right) \mathbb{T}\right] \tag{S6b}$$

whose derivation stems, respectively, from the work done by the total ($\mathbf{E}^{\text{t}}$) and scattered ($\mathbf{E}^{\text{s}}$) fields over the polarization current, $\mathbf{J}^{\text{s}}$. Specifically, we consider the following relations:[4]

$$\mathbf{J}^{\text{s}} = \frac{i}{k_0 Z_0}\mathbb{T}\mathbf{E}^{\text{i}} \quad \text{and} \quad \mathbf{E}^{\text{s}} = \mathbb{G}_0\mathbb{T}\mathbf{E}^{\text{i}} \tag{S7}$$

The absorption and scattering cross sections [Main text, Eqs. (1a) and (1b), respectively] are given respectively by,[5] $C_{\text{abs}} = P_{\text{abs}}/\frac{|E_0|^2}{2Z_0}$, $C_{\text{sca}} = P_{\text{sca}}/\frac{|E_0|^2}{2Z_0}$.

As discussed in the main text, to compute $\langle C_{\text{abs}}\rangle$ and $\langle C_{\text{sca}}\rangle$, we need an expression for $\langle \mathbf{E}^{\text{i}} \otimes \mathbf{E}^{\text{i}\dagger}\rangle$. For an incident field of the form $\mathbf{E}^{\text{i}} = E_0 e^{ik_0\hat{\mathbf{k}}^{\text{i}}(\mathbf{r}-\mathbf{r}_0)}$, where $\mathbf{r}_0$ represent the origin of the field away from the object, and considering the two orthogonal polarization $\hat{s}$ and $\hat{p}$:

$$\begin{aligned}
\langle \mathbf{E}^{\text{i}} \otimes \mathbf{E}^{\text{i}\dagger}\rangle &= \frac{1}{4\pi}\int_{4\pi} d\hat{\mathbf{k}}^{\text{i}}\ |E_0|^2\left[\hat{s}\hat{s} + \hat{p}\hat{p}\right] e^{ik_0\hat{\mathbf{k}}^{\text{i}}\cdot(\mathbf{r}-\mathbf{r}')} \\
&= |E_0|^2\frac{1}{4\pi}\int_{4\pi} d\hat{\mathbf{k}}^{\text{i}}\ \left[\hat{s}\hat{s} + \hat{p}\hat{p}\right]\frac{1}{2}\left[e^{ik_0\hat{\mathbf{k}}^{\text{i}}\cdot(\mathbf{r}-\mathbf{r}')} + e^{-ik_0\hat{\mathbf{k}}^{\text{i}}\cdot(\mathbf{r}-\mathbf{r}')}\right] \\
&= |E_0|^2\frac{1}{4\pi}\int_{4\pi} d\hat{\mathbf{k}}^{\text{i}}\left[\hat{s}\hat{s} + \hat{p}\hat{p}\right]\frac{1}{k_0^2}\int_0^{\infty} k_r^2 dk_r\ \frac{1}{2}\left[\delta\left(k_r - k_0\right) + \delta\left(k_r + k_0\right)\right] e^{ik_r\hat{\mathbf{k}}^{\text{i}}\cdot(\mathbf{r}-\mathbf{r}')} \\
&= |E_0|^2\frac{2\pi}{k_0}\frac{1}{(2\pi)^3}\int_{-\infty}^{\infty} d^3\mathbf{k}\ \left[\hat{s}\hat{s} + \hat{p}\hat{p}\right]\text{Im}\left(\frac{1}{k^2 - k_0^2}\right) e^{i\mathbf{k}\cdot(\mathbf{r}-\mathbf{r}')} \\
&= |E_0|^2\frac{2\pi}{k_0}\text{Asym}\left(\frac{1}{(2\pi)^3}\int_{-\infty}^{\infty} d^3\mathbf{k}\ \left[\mathbb{I} - \hat{k}\hat{k}\right]\frac{e^{i\mathbf{k}\cdot(\mathbf{r}-\mathbf{r}')}}{k^2 - k_0^2}\right) \\
&= |E_0|^2\frac{2\pi}{k_0}\text{Asym}\left(\mathbb{G}_0\right),
\end{aligned} \tag{S8}$$

where the second step exploits the reciprocity of $e^{ik_0\hat{\mathbf{k}}^{\text{i}}\cdot(\mathbf{r}-\mathbf{r}')}$, and the fourth step considers the identity,[6]

$$\frac{1}{2}\left[\delta\left(k_r - k_0\right) + \delta\left(k_r + k_0\right)\right] = \frac{k_0}{\pi}\text{Im}\left(\frac{1}{k^2 - k_0^2}\right).$$

The penultimate expression corresponds to the plane-wave representation of the Dyadic Green function.[2] Substitution of Eq. (S8) into Eqs. (1a) and (1b) (Main text), respectively, leads the relations Eqs. (4a) and (4b) in the Main text.

The asymmetry parameter is given by the force from scattering fields, $\mathbf{F}^{\mathrm{s}}$, through the relation:[7]

$$\mu_{\mathrm{sca}} C_{\mathrm{sca}} = \frac{\omega}{k_0 I_0} \hat{\mathbf{k}}^{\mathrm{i}} \cdot \mathbf{F}^{\mathrm{s}} \tag{S9}$$

The projection of the scattering force, $\hat{\mathbf{k}}^{\mathrm{i}} \cdot \mathbf{F}^{\mathrm{s}}$, expressed in terms of the induced currents is given by:[1,8]

$$\begin{aligned} \hat{\mathbf{k}}^{\mathrm{i}} \cdot \mathbf{F}^{\mathrm{s}} &= \hat{\mathbf{k}}^{\mathrm{i}} \cdot \frac{1}{2\omega} \mathrm{Im} \langle \mathbf{J}^{\mathrm{s}}, \nabla \mathbf{E}^{\mathrm{s}} \rangle \\ &= -\frac{1}{2\omega k_0 Z_0} \sum_j \mathrm{Tr} \left[\hat{\mathbf{k}}^{\mathrm{i}}_j \left(\mathbf{E}^{\mathrm{i}} \otimes \mathbf{E}^{\mathrm{i}\dagger} \right) \mathbb{T}^{\dagger} \mathrm{Sym} \left(\partial_j \mathbb{G}_0 \right) \mathbb{T} \right], \end{aligned} \tag{S10}$$

where we apply the relations from Eq. (S7).

Eq. (S10) represents a generalized form of the asymmetry parameter, regardless of the form of the source fields. Similarly to Eqs. (S6a) and (S6b), computation of the orientation average of, $\hat{\mathbf{k}}^{\mathrm{i}} \cdot \mathbf{F}^{\mathrm{s}}$, requires a direct expression for, $\left\langle \hat{\mathbf{k}}^{\mathrm{i}}_j \left(\mathbf{E}^{\mathrm{i}} \otimes \mathbf{E}^{\mathrm{i}\dagger} \right) \right\rangle$:

$$\begin{aligned} \left\langle \hat{\mathbf{k}}^{\mathrm{i}}_j \left(\mathbf{E}^{\mathrm{i}} \otimes \mathbf{E}^{\mathrm{i}\dagger} \right) \right\rangle &= \frac{1}{4\pi} \int_{4\pi} d\Omega \; \hat{\mathbf{k}}^{\mathrm{i}}_j \; (\hat{s}\hat{s} + \hat{p}\hat{p}) \, |E_0|^2 e^{i k_0 \hat{\mathbf{k}}^{\mathrm{i}} \cdot (\mathbf{r} - \mathbf{r}')} \\ &= |E_0|^2 \frac{2\pi}{i k_0^2} \partial_j \mathrm{Asym} \left(\mathbb{G}_0 \right) \\ &= |E_0|^2 \frac{2\pi}{i k_0^2} \frac{\partial_j \mathbb{G}_0(\mathbf{r}, \mathbf{r}') - \partial_j \mathbb{G}_0^{\dagger}(\mathbf{r}', \mathbf{r})}{2i} \\ &= |E_0|^2 \frac{2\pi}{i k_0^2} \frac{\partial_j \mathbb{G}_0(\mathbf{r}, \mathbf{r}') + \partial'_j \mathbb{G}_0^{\dagger}(\mathbf{r}', \mathbf{r})}{2i} \\ &= -|E_0|^2 \frac{2\pi}{k_0^2} \mathrm{Sym} \left(\partial_j \mathbb{G}_0 \right), \end{aligned} \tag{S11}$$

where, in the third step, we consider the identity $\partial'_j \mathbb{G}_0^{\dagger}(\mathbf{r}', \mathbf{r}) = -\partial_j \mathbb{G}_0^{\dagger}(\mathbf{r}', \mathbf{r})$.[9] Substitution

of Eqs. (S11) and (S10) into Eq. (S9), together with the relation $\langle\mu_{\rm sca}\rangle = \frac{\langle\mu_{\rm sca}C_{\rm abs}\rangle}{\langle C_{\rm sca}\rangle}$ leads to the expression Eq. (4c) in the main text.

The expression Eqs. (4a), (4b) and (4c) in the Main text, can be extended for linear, homogeneous and non-absorbing external medium, by replacing k_0, for n_0k_0, where n_0 is the refractive index of the external medium.

1.2 Surface-current BEM formulation

Particularly for the BEM, under the surface-current formulation, the fields and currents on an object n are, respectively, represented by the bi-linear expressions[10,11]

$$\boldsymbol{\phi}_n = \begin{Bmatrix} \mathbf{E} \\ \mathbf{H} \end{Bmatrix} \quad \text{and} \quad \boldsymbol{\xi}_n = \begin{Bmatrix} \mathbf{K} \\ \mathbf{N} \end{Bmatrix},$$

where $\mathbf{K}$ and $\mathbf{N}$ are the surface electric and magnetic current, respectively.

Using bi-linear expressions, the electromagnetic field is given by $\boldsymbol{\phi}_n = \mathbb{\Gamma}_n\boldsymbol{\xi}_n$. In this formula,

$$\mathbb{\Gamma}_n = ik_n \begin{bmatrix} Z_n\mathbb{G}_n & \mathbb{C}_n \\ -\mathbb{C}_n & \frac{1}{Z_n}\mathbb{G}_n \end{bmatrix}, \tag{S12}$$

where $\mathbb{C}_n = \frac{i}{k_n}\nabla\times\mathbb{G}_n$, and k_n and Z_n are the wavevector and impedance of medium n, respectively.

Eq. (S22) in its bi-linear form is now expressed as:

$$\left[\mathbb{H}_0 - ik_0\mathbf{Z}_0^{-1}\mathbb{I} - \sum_n \mathbb{V}_n\right]\boldsymbol{\phi}^t = 0,$$

where $\mathbb{H}_0 = \begin{bmatrix} 0 & \nabla\times \\ -\nabla\times & 0 \end{bmatrix}$, $\mathbf{Z}_0^{-1} = \begin{bmatrix} Z_0^{-1} & 0 \\ 0 & Z_0 \end{bmatrix}$, and $\mathbb{V}_n = ik_0\mathbf{Z}_0^{-1}\begin{bmatrix} \varepsilon_n - 1 & 0 \\ 0 & \mu_n - 1 \end{bmatrix}$. Notice that in this case, the elements of $\mathbf{V}$ and $\mathbf{G}^0$, are respectively given by $\mathbb{V}_{nm} = \delta_{nm}\mathbb{\Gamma}_n^{-1}$, and $G^0_{nm} = \mathbb{\Gamma}_0(\mathbf{r}_n, \mathbf{r}_m)$

The solution is obtained by expanding the surface currents on a particular basis, $\mathbf{f}_r^n(\mathbf{r})$, as: $\xi_n(\mathbf{r}) = \sum_r x_r^n \mathbf{f}_r^n(\mathbf{r})$, where the expansion elements x_r^n are obtained from boundary conditions. Further details can be found elsewhere.[10,11]

From the surface current expansion, the operators-based form of Eqs. (S25a), (S25b) and (S25c) is now given in matrix notations by:

$$\langle C_{\text{abs}} \rangle = \frac{2\pi}{k_0^2} \text{Tr}\left[\text{Sym}\left(G^0\right) W^\dagger \; \text{Sym}\left(-G^{\text{in}}\right) W\right] \tag{S13a}$$

$$\langle C_{\text{sca}} \rangle = \frac{2\pi}{k_0^2} \text{Tr}\left[\text{Sym}\left(G^0\right) W^\dagger \; \text{Sym}\left(G^0\right) W\right] \tag{S13b}$$

$$\langle \mu_{\text{sca}} C_{\text{sca}} \rangle = \frac{2\pi}{k_0^4} \sum_l \text{Tr}\left[\text{Asym}\left(\partial_l G^0\right) W^\dagger \; \text{Asym}\left(\partial_l G^0\right) W\right], \tag{S13c}$$

where the elements of the G^0 and G^{in} are respectively given by $G_{ij}^{0,nm} = \langle \mathbf{f}_i^n, \mathbb{\Gamma}_0 \mathbf{f}_j^m \rangle$ and $G_{ij}^{\text{in},nm} = \delta_{nm} \langle \mathbf{f}_i^n, \mathbb{\Gamma}_m \mathbf{f}_j^m \rangle$, and $W = [G^{\text{in}} - G^0]$. In this notation, the operator $\langle \, , \, \rangle$, denotes the conjugated inner product: $\langle \mathbf{u}, \mathbf{v} \rangle = \int d^3r \; \mathbf{u}^\dagger(\mathbf{r}) \cdot \mathbf{v}(\mathbf{r})$, where $\mathbf{u}$ and $\mathbf{v}$ are vector fields.

Note that due to the imaginary prefactor in Eq. (S12), the operators "Sym ()" and "Asym ()" in Eqs. (S13a), (S13b) and (S13c) are in opposite order in comparison with Eqs. (S25a), (S25b) and (S25c).

a

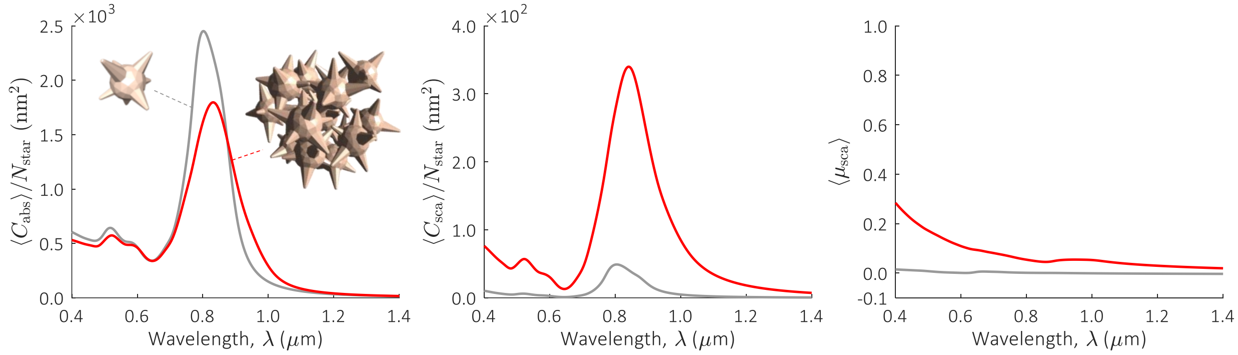

b

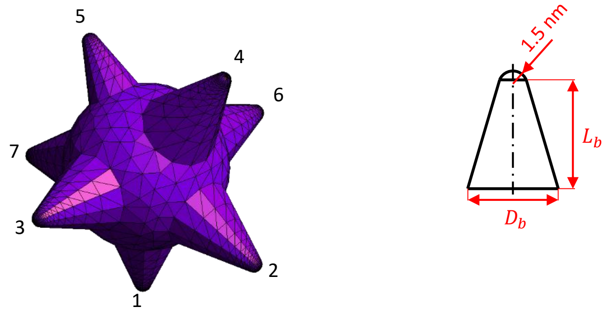

Branch No	L_b (nm)	D_b (nm)
1	9	9
2	14	10
3	13	11
4	17	11
5	9	6
6	14	11
7	15	12

Figure S1: **Average light scattering of agglomerated gold nanostars (a)** $\langle C_{\text{abs}}\rangle$, $\langle C_{\text{sca}}\rangle$ and $\langle \mu_{\text{sca}}\rangle$ of a single gold nanostar (grey) and cluster of agglomerated gold nanostars (red). The curves $\langle C_{\text{abs}}\rangle$ and $\langle C_{\text{sca}}\rangle$ are normalized to the number of stars (N_{star}) for direct comparison. The cluster is based on 15 stars at 20% volume fraction. **(b)** Model of the star considered for the simulations. The model consist on a star with 7 legs with the dimensions indicated by the table at the right-hand side of the figure. All the simulations were performed by the BEM code for average light scattering simulations.[12] The refractive index of gold can be found elsewhere.[13]

Eqs. (S13a), (S13b) and (S13c) were implemented into a BEM application for average light scattering simulations.[12] The flexibility of the BEM algorithm enables to study objects of arbitrary morphology.[10] As a demonstration, we simulated the average light scattering parameters $\langle C_{\text{abs}}\rangle$, $\langle C_{\text{sca}}\rangle$ and $\langle \mu_{\text{sca}}\rangle$ of a single gold nanostar and a cluster of agglomerated gold nanostars (Fig. S1). Fig. S1(a) illustrates the effects of interparticle coupling and interference of the scattered fields from the stars in cluster, which results in a reduction of $\langle C_{\text{abs}}\rangle/N_{\text{star}}$ and an enhancement of $\langle C_{\text{sca}}\rangle/N_{\text{star}}$ in comparison with a single star. Similarly, the scattering anisotropy is also clearly affected in the star cluster, as indicated by the variations in $\langle \mu_{\text{sca}}\rangle$. Fig. S1(b) shows the dimensions of the gold nanostar model considered

for the simulations.

1.3 Average light scattering in Spherical wave basis: T-matrix formulation

The operator $\mathbb{T}$ for spherical wave basis is given by:[1]

$$\mathbb{T}(\mathbf{r},\mathbf{r}') = i\sum_{Plm}\sum_{P'l'm'} \mathbf{E}^{\text{reg}}_{Plm}(\mathbf{r}) T^{PP'}_{lm,l'm'} \mathbf{E}^{\text{reg}}_{P'l'-m'}(\mathbf{r}') \tag{S14}$$

where $P = M, N$ correspond to the two orthogonal polarizations, $l = 1, 2...\infty$ and $m = -l, ..., 0, ..., +l$; $T^{PP'}_{lm,l'm'}$ are the elements of the T-matrix,[2] and $\mathbf{E}^{\text{reg}}_{Plm}(\mathbf{r})$ are the spherical waves regular at the origin, defined at the spherical coordinates r, θ and ϕ:[1]

$$\begin{aligned}
\mathbf{E}^{\text{reg}}_{Mlm}(\mathbf{r}) &= \sqrt{(-1)^m k_0}\frac{1}{\sqrt{l(l+1)}} j_l\left(k_0 r\right) \nabla \times Y^m_l(\theta,\phi), \\
\mathbf{E}^{\text{reg}}_{Nlm}(\mathbf{r}) &= k_0 \nabla \times \mathbf{E}^{\text{reg}}_{Mlm}(\mathbf{r}),
\end{aligned}$$

j_l is the spherical Bessel function of order l, and $Y^m_l(\theta,\phi)$ are the spherical harmonics according to the definition from Ref. [14]. Note that $\mathbf{E}^{\text{reg}*}_{Plm}(\mathbf{r}) = \mathbf{E}^{\text{reg}}_{Nl-m}(\mathbf{r})$, where * is the complex conjugate.[1]

Because $\mathbb{G}_0$ is a symmetric and reciprocal dyadic, $\text{Asym}\left(\mathbb{G}_0\right) = \text{Im}\left(\mathbb{G}_0\right)$.[9] For spherical wave basis, it can be demonstrated that:[1]

$$\text{Im}\left(\mathbb{G}_0\right) = \sum_{Plm} \mathbf{E}^{\text{reg}}_{Plm}(\mathbf{r}) \otimes \mathbf{E}^{\text{reg}}_{Pl-m}(\mathbf{r}') \tag{S15}$$

Replacing Eqs. (S14) and (S15) into Eq. (4a) and (4b) (Main text), we derive the expres-

sions of $\langle C_{\mathrm{abs}} \rangle$ and $\langle C_{\mathrm{sca}} \rangle$ for T-matrix, i.e.:[15,16]

$$\langle C_{\mathrm{abs}} \rangle = \frac{2\pi}{k_0^2} \sum_{Plm} \sum_{P'l'm'} \left[\mathrm{Re}\left(T^{PP'}_{lm,l'm'} \right) - |T^{PP'}_{lm,l'm'}|^2 \right] \tag{S16}$$

$$\langle C_{\mathrm{sca}} \rangle = \frac{2\pi}{k_0^2} \sum_{Plm} \sum_{P'l'm'} |T^{PP'}_{lm,l'm'}|^2 \tag{S17}$$

where in the $\langle C_{\mathrm{abs}} \rangle$ formula, we consider the relation $\mathbb{T}^\dagger \mathrm{Asym}\left(-\mathbb{V}^{-1}\right) \mathbb{T} = \mathrm{Asym}\left(\mathbb{T}\right) - \mathbb{T}^\dagger \mathrm{Asym}\left(\mathbb{G}_0\right) \mathbb{T}$.

To derive the T-Matrix formula of $\langle \mu_{\mathrm{sca}} \rangle$, we use the relation: $\mathrm{Sym}\left(\partial_j \mathbb{G}_0\right) = \partial_j \mathrm{Im}\left(\mathbb{G}_0\right)$; which stems form the reciprocal and anti-symmetric properties of $\partial_j \mathbb{G}_0$.[9] For spherical waves basis, this expression is:[1]

$$\partial_j \mathrm{Im}\left(\mathbb{G}_0\right) = -\sum_{Plm} \sum_{P'l'm'} \mathbf{p_j}^{PP'}_{lm,'l'm'} \mathbf{E}^{\mathrm{reg}}_{Plm}(\mathbf{r}) \otimes \mathbf{E}^{\mathrm{reg}}_{P'l'-m'}(\mathbf{r'}) \tag{S18}$$

where $\mathbf{p_j}^{PP'}_{lm,'l'm'} = \partial_j \mathcal{V}^{PP'}_{lm,l'm'}(a)|_{a=0}$, and $\mathcal{V}$ is the translation operator of regular waves.[17] The form of $\mathcal{V}$ for spherical waves is given in Ref. [17], Appendix C.3.

By replacing Eqs. (S14) and (S18) into Eq. (4c) of the Main text, we derive the T-matrix formulation of $\langle \mu_{\mathrm{sca}} \rangle$:

$$\langle \mu_{\mathrm{sca}} \rangle = \frac{1}{\langle C_{\mathrm{sca}} \rangle} \frac{2\pi}{k_0^2} \sum_{j=x,y,z} \sum_{Plm} \sum_{P'l'm'} \sum_{P''l''m''} \mathbf{p_j}^{PP'}_{lm,'l'm'} \; T^{*P'P''}_{l'm',l''m''} \mathbf{p_j}^{P''P'''}_{l''m'','l'''m'''} \; T^{P'''P}_{l'''m''',lm} \tag{S19}$$

For a spherical object, Eq. (S19) can be simplified to:[1,18]

$$\langle \mu_{\mathrm{sca}} \rangle = \frac{1}{\langle C_{\mathrm{sca}} \rangle} \frac{2\pi}{k_0^2} \sum_{Plm} \mathrm{Re}\left[3a(l,m)^2 T^P_l T^{\bar{P}*}_l + 6b(l,m)^2 T^P_l T^{P*}_{l+1} \right] \tag{S20}$$

where $\bar{P} = N$ if $P = M$ and vice versa, T_l^P are the mie-scattering coefficients,[5] and:

$$a(l,m) = \frac{m}{l(l+1)}$$
$$b(l,m) = \frac{1}{l+1}\sqrt{\frac{l(l+2)(l-m+1)(l+m+1)}{(2l+1)(2l+3)}}$$

Using the identities $\sum_{m=-l}^{l} a(n,m)^2 = \frac{2l+1}{3l(l+1)}$ and $\sum_{m=-l}^{l} b(n,m)^2 = \frac{l(l+2)}{3(l+1)}$, and noting that $\mathrm{Re}\left(T_l^M T_l^{N*}\right) = \mathrm{Re}\left(T_l^N T_l^{M*}\right)$, we can simplify Eq. (S20) to derive the well-known formula:[5]

$$\langle \mu_{\mathrm{sca}} \rangle = \frac{1}{\langle C_{\mathrm{sca}} \rangle} \frac{4\pi}{k_0^2} \sum_l \mathrm{Re}\left[\frac{2l+1}{l(l+1)} T_l^M T_l^{N*} + \frac{l(l+2)}{(l+1)}\left(T_l^M T_{l+1}^{M*} + T_l^N T_{l+1}^{N*}\right)\right] \tag{S21}$$

1.4 Formulation for multiple objects

In the case of multiple objects Eq. (S1), becomes:

$$\left[\mathbb{H}_0 - k_0^2 \mathbb{I} - \sum_{n=1}^{N} \mathbb{V}_n\right] \mathbf{E}^{\mathrm{t}} = 0, \tag{S22}$$

where N is the total number of objects. The solution of this equation is given by:[1] $\mathbf{E}^{\mathrm{t}} = \mathbf{E}^{\mathrm{i}} + \sum_n \mathbb{G}_0 \mathbb{T}_n \mathbf{E}_n^{\mathrm{i}}$, and in matrix form:

$$\boldsymbol{\phi}^{\mathrm{t}} = \boldsymbol{\phi}^{\mathrm{i}} + \mathbf{G}^0 \mathbf{W} \boldsymbol{\phi}^{\mathrm{i}} \tag{S23}$$

where $\boldsymbol{\phi}^{\mathrm{i}}$ is a column vector, whose elements contain the incident fields on each object, $\phi_n^{\mathrm{i}} = \mathbf{E}_n^{\mathrm{i}}$; $\mathbf{W}^{-1} = \mathbf{G}_0 - \mathbf{V}^{-1}$ is the analogous of $\mathbb{T}$ for a cluster; $\mathbf{V}$ is a band matrix operator whose elements are $\mathbb{V}_{nm} = \mathbb{V}_m \delta_{nm}$; and the matrix operator $\mathbf{G}^0$ represents the interaction between the objects in the free space, whose elements are $\mathbb{G}_{nm}^0 = \mathbb{G}_0\left(\omega; \mathbf{r}_n, \mathbf{r}_m\right)$. The rest of the elements are defined in the main text.

Following the matrix notation, we extend the relations from Eq. (S7) to a vector form in

terms of the induced current, $\boldsymbol{\xi}^{\mathrm{s}}$, and fields, $\boldsymbol{\phi}^{\mathrm{s}}$:

$$\boldsymbol{\xi}^{\mathrm{s}} = -\frac{i}{k_0 Z_0}\mathbf{V}\boldsymbol{\phi}^{\mathrm{t}} \quad \text{and} \quad \boldsymbol{\phi}^{\mathrm{s}} = \mathbf{G}^0\mathbf{W}\boldsymbol{\phi}^{\mathrm{i}}. \tag{S24}$$

Analogous to the derivation of $\langle C_{\mathrm{abs}}\rangle$, $\langle C_{\mathrm{sca}}\rangle$ and $\langle \mu_{\mathrm{sca}}\rangle$ for a single object, we use Eq. (S23) and the relations in Eq. (S24), to derive:

$$\langle C_{\mathrm{abs}}\rangle = \frac{2\pi}{k_0^2}\mathrm{Tr}\left[\mathbf{W}\,\mathrm{Asym}\left(\mathbf{G}^0\right)\mathbf{W}^\dagger \mathrm{Asym}\left(-\mathbf{V}^{-1}\right)\right], \tag{S25a}$$

$$\langle C_{\mathrm{sca}}\rangle = \frac{2\pi}{k_0^2}\mathrm{Tr}\left[\mathbf{W}\,\mathrm{Asym}\left(\mathbf{G}^0\right)\mathbf{W}^\dagger \mathrm{Asym}\left(\mathbf{G}^0\right)\right], \tag{S25b}$$

$$\langle \mu_{\mathrm{sca}}\rangle = \frac{1}{\langle C_{\mathrm{sca}}\rangle}\frac{2\pi}{k_0^4}\sum_j \mathrm{Tr}\left[\mathbf{W}\,\mathrm{Sym}\left(\partial_j\mathbf{G}^0\right)\mathbf{W}^\dagger \mathrm{Sym}\left(\partial_j\mathbf{G}^0\right)\right], \tag{S25c}$$

these expressions represent the most general form of the average light scattering theory. They can be applied to single and group of particles, including heterogeneous collections of particles.

1.5 Formulation for an individual object in a cluster

Using Eqs. (S25a), (S25b) and (S25c), we deduce the individual contribution of an object n in the cluster:

$$\langle C^n_{\mathrm{abs}}\rangle = \frac{2\pi}{k_0^2}\mathrm{Tr}\left[\mathbb{S}_{nn}\,\mathrm{Asym}\left(-\mathbb{V}_n^{-1}\right)\right] \tag{S26a}$$

$$\langle C^n_{\mathrm{sca}}\rangle = \frac{2\pi}{k_0^2}\left\{\mathrm{Tr}\left[\mathbb{S}_{nn}\,\mathrm{Asym}\left(\mathbb{G}^0_{nn}\right)\right] + \sum_{n\neq m}^{N}\mathrm{Tr}\left[\mathbb{S}_{nm}\,\mathrm{Asym}\left(\mathbb{G}^0_{nm}\right)\right]\right\} \tag{S26b}$$

$$\langle \mu^n_{\mathrm{sca}}\rangle = \frac{N}{\langle C_{\mathrm{sca}}\rangle}\frac{2\pi}{k_0^4}\sum_j\left\{\mathrm{Tr}\left[\mathbb{S}^{\nabla}_{j,nn}\,\mathrm{Sym}\left(\partial_j\mathbb{G}^0_{nn}\right)\right] + \sum_{n\neq m}^{N}\mathrm{Tr}\left[\mathbb{S}^{\nabla}_{j,nm}\,\mathrm{Sym}\left(\partial_j\mathbb{G}^0_{nm}\right)\right]\right\}, \tag{S26c}$$

where $\mathbf{S} = \left[\mathbf{W}\,\mathrm{Asym}\left(\mathbf{G}^0\right)\mathbf{W}^\dagger\right]$, and $\mathbf{S}^{\nabla}_j = \left[\mathbf{W}\,\mathrm{Sym}\left(\partial_j\mathbf{G}^0\right)\mathbf{W}^\dagger\right]$.

In Eqs. (S26b) and (S26c) the first and second term inside the curly brackets represent, self and interference scattering, respectively.[19] Note that $\langle \mu^n_{\mathrm{sca}}\rangle$ in Eq. (S26c) is scaled by N

for better comparison with $\langle \mu_{\text{sca}} \rangle$ from an isolated object.

To recover the total values of the cluster [Eqs. (S25a), (S25b) and (S25c)]:

$$\langle C_{\text{abs}} \rangle = \sum_{n=1}^{N} \langle C_{\text{abs}}^{n} \rangle$$
$$\langle C_{\text{sca}} \rangle = \sum_{n=1}^{N} \langle C_{\text{sca}}^{n} \rangle$$
$$\langle \mu_{\text{sca}} \rangle = \frac{1}{N} \sum_{n=1}^{N} \langle \mu_{\text{sca}}^{n} \rangle.$$

1.6 Approximation for subwavelength particles

For small objects, $\text{Asym}\left(\mathbb{G}_0\right) \approx \frac{k_0}{6\pi}\mathbb{I}$,[20] and, $\mathbb{T} \approx 4\pi k_0 \boldsymbol{\alpha}$,[a] where $\boldsymbol{\alpha}$ is the polarizability tensor. Substitution into Eq. (S26a), together with the relation

$$\mathbb{T}^{\dagger}\text{Asym}\left(-\mathbb{V}^{-1}\right)\mathbb{T} = \text{Asym}\left(\mathbb{T}\right) - \mathbb{T}^{\dagger}\text{Asym}\left(\mathbb{G}_0\right)\mathbb{T},$$

gives:

$$\langle C_{\text{abs}} \rangle = \frac{4\pi}{3} k_0 \ \text{Tr}\left[\text{Im}\left(\boldsymbol{\alpha}\right)\right] - \frac{8\pi}{9} k_0^4 \ \text{Tr}\left[\boldsymbol{\alpha}^{\dagger}\boldsymbol{\alpha}\right]. \tag{S27}$$

Because the particles are small, the second term in Eq. (S27) is negligible in comparison with the first, leading to

$$\langle C_{\text{abs}} \rangle \approx \frac{1}{3}\left(C_{\text{abs},x} + C_{\text{abs},y} + C_{\text{abs},z}\right).$$

The second term in Eq. (S27) corresponds to the scattering of the particle, which can be written as:

$$\langle C_{\text{sca}} \rangle = \frac{8\pi}{9} k_0^4 \ \sum_{ij} |\alpha_{ij}|^2,$$

where α_{ij} are the elements of the tensor $\boldsymbol{\alpha}$. If $\boldsymbol{\alpha}$ is a diagonal tensor, $\text{Tr}\left[\boldsymbol{\alpha}^{\dagger}\boldsymbol{\alpha}\right] = |\alpha_{xx}|^2 +$

[a] the expression is deducted from the electric field generated by a point dipole[14]

$|\alpha_{yy}|^2 + |\alpha_{zz}|^2$, and we derive the commonly used relation,[21,22]

$$\langle C_{\mathrm{sca}} \rangle \approx \frac{1}{3} \left(C_{\mathrm{sca},x} + C_{\mathrm{sca},y} + C_{\mathrm{sca},z} \right).$$

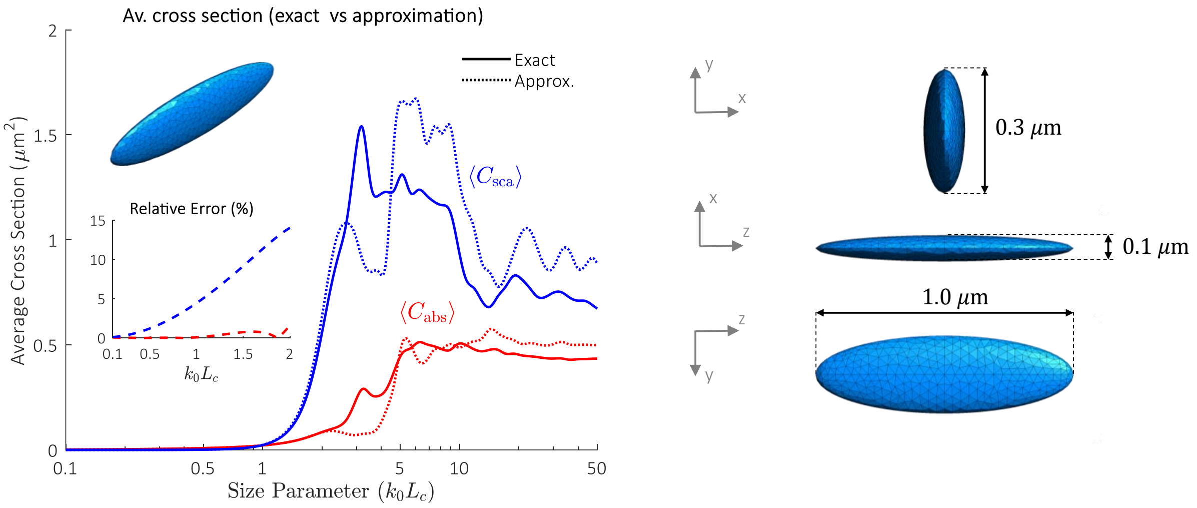

Figure S2: **Average light scattering of randomly oriented particles of subwavelength size. Comparison of exact solution and approximations** The figure shows $\langle C_{\mathrm{abs}} \rangle$ and $\langle C_{\mathrm{sca}} \rangle$ of a randomly oriented ellipsoid plotted against the size parameter k_0L_c ($L_c = 1.0$ μm). The exact solution for $\langle C_{\mathrm{abs}} \rangle$ and $\langle C_{\mathrm{sca}} \rangle$ is based on Eqs. (4a) and (4b) (Main text), respectively, and was computed by our BEM application for average light scattering simulations.[12] The approximated results are based on the relations, $\langle C_{\mathrm{abs}} \rangle \approx \frac{1}{3} \left(C_{\mathrm{abs},x} + C_{\mathrm{abs},y} + C_{\mathrm{abs},z} \right)$ and $\langle C_{\mathrm{sca}} \rangle \approx \frac{1}{3} \left(C_{\mathrm{sca},x} + C_{\mathrm{sca},y} + C_{\mathrm{sca},z} \right)$. The inset shows the relative error between the approximated and the exact solutions for small size parameters. The dimensions of the ellipsoid are indicated at the right figure. The refractive index of the ellipsoid is $N = 3.5 + 0.1i$.

In Fig. S2, we compute $\langle C_{\mathrm{abs}} \rangle$ and $\langle C_{\mathrm{sca}} \rangle$ for a randomly oriented ellipsoid, using the exact solution [Main text, Eqs. (4a) and (4b), respectively] and the approximation for small objects. The ellipsoid represents a typical case where $\boldsymbol{\alpha}$ is not diagonal. As shown in the inset of Fig. S2, the approximation $\langle C_{\mathrm{sca}} \rangle \approx \frac{1}{3} \left(C_{\mathrm{sca},x} + C_{\mathrm{sca},y} + C_{\mathrm{sca},z} \right)$, induces considerable error, even when k_0L_c is small. On the other hand, the approximation for $\langle C_{\mathrm{abs}} \rangle$, shows a small relative error ($< 2\%$) for small particles ($k_0L_c < 2$). For larger particles ($k_0L_c > 2$), both approximations fail.

2 Average light scattering from fluctuational electrodynamics

2.1 Vacuum friction and averaged light scattering

An isolated object moving at a small velocity $\mathbf{v}$ experiences a non-conservative friction force, $\mathbf{F}_f$, that results from the interaction with thermal fluctuations in the free space. To a first order approximation, the vacuum friction is given by, $\mathbf{F}_f = -\hat{\gamma} \cdot \mathbf{v}$, where $\hat{\gamma}$ is the friction tensor given by:[18]

$$\hat{\gamma}_{ij} = \frac{2\hbar^2}{\pi k_\mathrm{B} T} \int_0^\infty d\omega \frac{e^{\hbar\omega/k_\mathrm{B}T}}{\left(e^{\hbar\omega/k_\mathrm{B}T} - 1\right)^2} \mathrm{Im}\{\mathrm{Tr}\left[\partial_i \left(1 + \mathbb{G}_0 \mathbb{T}\right) \partial_j \mathrm{Asym}\left(\mathbb{G}_0\right) \mathbb{T}^\dagger\right]\}, \tag{S28}$$

here, k_B is the Boltzmann constant, and T is the equilibrium temperature of the system. Integrating the vacuum friction force over all solid angles leads to the relation,[23] $\langle \mathbf{F}_f \rangle = \gamma \mathbf{v}$, where $\gamma = \sum_i \hat{\gamma}_{ii}$ is the friction coefficient.

From the relations, $\partial_i \mathrm{Asym}\left(\mathbb{G}_0\right) = -i\mathrm{Sym}\left(\partial_i \mathbb{G}_0\right)$,[18] and, $\partial_i^2 \mathrm{Asym}\left(\mathbb{G}_0\right) = -k_0^2 \mathrm{Asym}\left(\mathbb{G}_0\right)$,[b] the friction coefficient is expressed as:

$$\begin{aligned}\gamma = \frac{2\hbar^2}{\pi k_\mathrm{B} T} \int_0^\infty d\omega \frac{e^{\hbar\omega/k_\mathrm{B}T}}{\left(e^{\hbar\omega/k_\mathrm{B}T} - 1\right)^2} &\{k_0^2 \mathrm{Tr}\left[\mathrm{Asym}\left(\mathbb{G}_0\right) \mathrm{Asym}\left(\mathbb{T}\right)\right] \\ &- \sum_i \mathrm{Tr}\left[\mathrm{Sym}\left(\partial_i \mathbb{G}_0\right) \mathbb{T}^\dagger\ \mathrm{Sym}\left(\partial_i \mathbb{G}_0\right) \mathbb{T}\right]\}.\end{aligned} \tag{S29}$$

Applying Eqs. (4a) and (4b) (Main text) into the first term inside the curly brackets,[c] and Eq. (4c) (Main text) into the second, we derive Eq. (6) from the main text.

[b] This expression is based on the fact that $\mathrm{Asym}\left(\mathbb{G}_0\right)$ can be expressed by propagating waves, as demonstrated in Eq. (S8)

[c] we consider the relation: $\mathrm{Asym}\left(\mathbb{T}\right) = -\mathbb{T}^\dagger \mathrm{Asym}\left(\mathbb{T}^{-1}\right) \mathbb{T} = \mathbb{T}^\dagger \mathrm{Asym}\left(\mathbb{G}_0 - \mathbb{V}^{-1}\right) \mathbb{T}$

3 Validation of average light scattering theory against analytical solutions

3.1 Average light scattering of randomly oriented spheroids

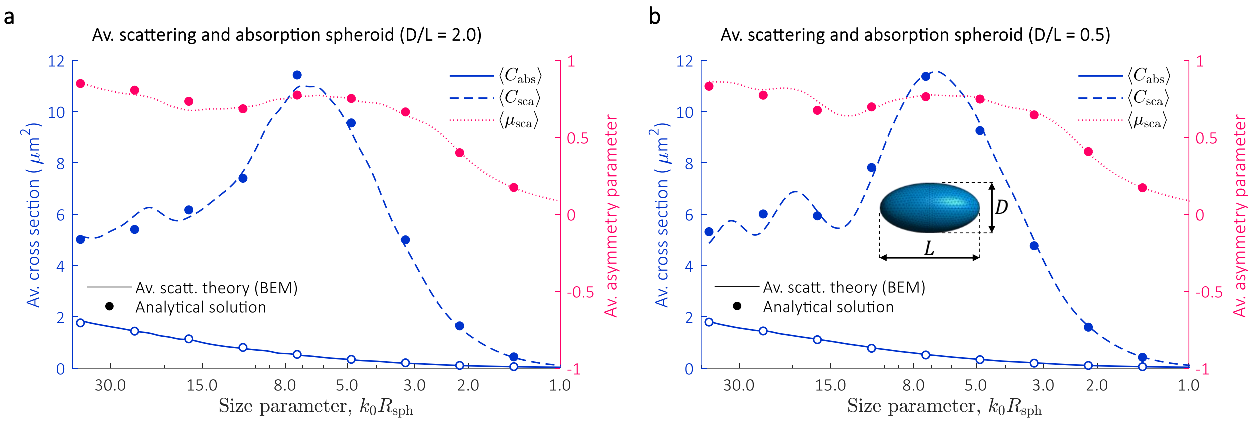

Figure S3: **Average light scattering of randomly oriented spheroids.** $\langle C_{\mathrm{abs}}\rangle$, $\langle C_{\mathrm{sca}}\rangle$ and $\langle \mu_{\mathrm{sca}}\rangle$ of **(a)** oblate ($D/L = 2.0$) and **(b)** prolate spheroids ($D/L = 0.5$), as a function of the size parameter $k_0 R_{\mathrm{sph}}$, where $R_{\mathrm{sph}} = 1$ is the radius of a sphere with equivalent surface area.[24] The results are computed by the BEM code for average light scattering simulations,[12] and compared with the analytical solutions.[25] The dielectric constant of the ellipsoids is $\varepsilon_p = 2.2499 + 0.0240i$. The principal and secondary axis of the oblate(prolate) spheroid are $L = 0.6016(1.5304)$ and $D = 1.2032(0.7653)$, respectively.

We validate our code for average scattering simulations[12] against the analytical solution for oblate [Fig. S3(a)] and prolate [Fig. S3(b)] spheroids.[25] In terms of relative error, the agreement of the simulations for the oblate(prolate) spheroid is 3.6%(1.6%), 4.5%(3.7%) and 2.6%(3.2%) for $\langle C_{\mathrm{abs}}\rangle$, $\langle C_{\mathrm{sca}}\rangle$ and $\langle \mu_{\mathrm{sca}}\rangle$, respectively. The discrepancy becomes more significant at short wavelengths, specifically at $L_{\max}/\lambda > 2.37(2.80)$ for the oblate(prolate) spheroid.

4 Validation of radiative transfer simulation against experiments

4.1 Characterization of as-grown VO_2(M) microcrystals

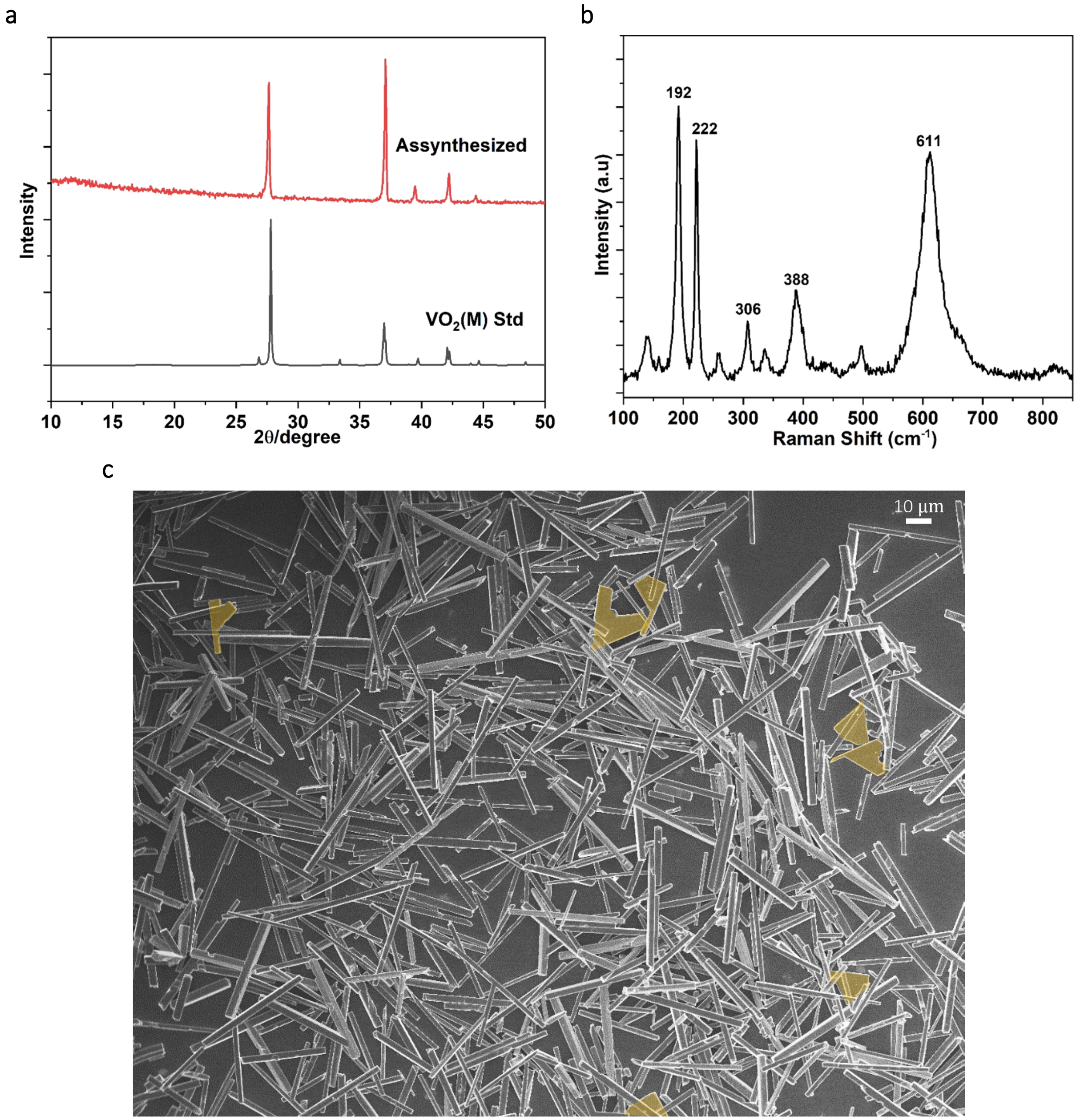

Figure S4: **Characterization of as-grown VO_2(M) powder.** **(a)** X-ray diffraction and **(b)** Raman shift spectroscopy of VO_2(M) microcrystals grown by hydrothermal synthesis (see Main Text, Materials and Methods). **(c)** SEM image of VO_2(M) powder used for the estimation of size distribution [Main text, Fig. 3(c)]. Most crystals have a bar morphology with few having a larger flat morphology (flakes), which are marked in yellow.

4.2 Average light scattering of VO_2(M) bars as a function of L.

The sensitivity of the VO_2(M) bars average light scattering to changes in L for $W = 2.5$ μmis shown in Fig. S5. As shown in the figure, $\langle C_{\text{abs}} \rangle$, $\langle C_{\text{sca}} \rangle$ and $\langle \mu_{\text{sca}} \rangle$ show small variation at $L > 15$ μm. We noted a similar response for other values of W (not shown here).

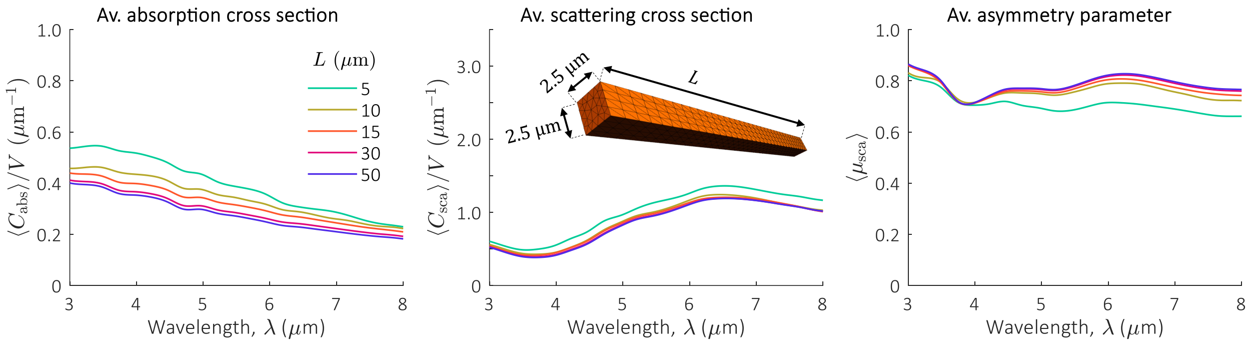

Figure S5: **$\langle C_{\text{abs}} \rangle$, $\langle C_{\text{sca}} \rangle$ and $\langle \mu_{\text{sca}} \rangle$ of VO_2(M) bars of variable length and fixed width.** The bars dimensions are: $W = 2.5$ μm and $L = 5$, 10, 15, 30 and 50 μm. The refractive index of the host, $n_{\text{host}} = 1.5$, and the refractive index of the VO_2(M) bars was obtained from the literature (see "film 2" in Wan et al[26]).

4.3 Estimation of average light scattering of VO_2(M) microcrystal ensemble

The average light scattering of the VO_2(M) microcrystal ensemble is calculated through:

$$\langle C_{\text{abs}} \rangle = \sum_{W,L} F_{W,L} \langle C_{\text{abs}} \rangle_{W,L} + F_{\text{flake}} \langle C_{\text{abs}} \rangle_{\text{flake}} \tag{S30a}$$

$$\langle C_{\text{sca}} \rangle = \sum_{W,L} F_{W,L} \langle C_{\text{sca}} \rangle_{W,L} + F_{\text{flake}} \langle C_{\text{sca}} \rangle_{\text{flake}} \tag{S30b}$$

$$\langle \mu_{\text{sca}} \rangle = \frac{1}{\langle C_{\text{sca}} \rangle} \left[\sum_{W,L} F_{W,L} \langle C_{\text{sca}} \rangle_{W,L} \langle \mu_{\text{sca}} \rangle_{W,L} + F_{\text{flake}} \langle C_{\text{sca}} \rangle_{\text{flake}} \langle \mu_{\text{sca}} \rangle_{\text{flake}} \right] \tag{S30c}$$

where F represent the number microcrystals of a particular morphology in the ensemble, and the subscripts "W, L" and "flake", indicate a microbars of dimensions L and W, and flakes, respectively. For $F_{W,L}$, we considered the estimated size distribution [Main text, Fig. 3(c)], while $F_{\text{flake}} = 5$, as an approximate of the number of flakes found in the sample.

The volume of the microcrystal ensemble V is given by:

$$V = \sum_{W,L} F_{W,L} V_{W,L} + 5V_{\text{flake}}, \tag{S31}$$

where $V_{W,L}$ is the volume of a single bar with dimensions W and L, and V_{flake} is the volume of the flake structure.

4.4 Prediction of refractive index and scattering from PE films

Overall, the optical properties of PE are highly dependent on the crystallinity and the manufacturing process.[27] Thus, we extracted the complex refractive index of a pure PE film (101 μm thick), n_{PE}, fabricated under the same conditions than those used for the composite films. First [Fig. S6(a)], T_{tot}, T_{spec} and R_{tot} of the PE film where measured by FTIR and a gold integrating sphere (Main text, Materials and Methods). The real part of n_{PE} was obtained from Palik et al.[27] The extinction coefficient, $\kappa_{\text{PE}} = \text{Im}\,(n_{\text{PE}})$, was extracted from the equation:[28]

$$T_{\text{tot}} = \frac{(1 - R_f)^2 \exp\left(-2k_0 t_{\text{film}} \kappa_{\text{PE}}\right)}{1 - R_f^2 \exp\left(-4k_0 t_{\text{film}} \kappa_{\text{PE}}\right)},$$

where t_{film} is the thickness of the PE film and R_f is the reflectance at the air/PE interface. R_f was estimated from Fresnel law and the refractive index of PE from Palik et al.[27]

As shown in Fig. S6(a), $T_{\text{tot}} \neq T_{\text{spec}}$ at most parts of the spectrum, indicating a small scattering component in the film. To include this features in the modeling, we assume a small concentration of scattering particles inside the film. Because $\kappa_{\text{PE}} \ll \text{Re}\,(n_{\text{PE}})$, $T_{\text{tot}} \approx (1 - R_f)^2 \exp\left(-2k_0 t_{\text{film}} \kappa_{\text{PE}}\right)$.[27] Additionally, at low particle concentrations,[29]

$$T_{\text{spec}} \approx (1 - R_f)^2 \exp\left[\left(-f_v C_{\text{sca}}/V_p - 2k_0 \kappa_{\text{PE}}\right) t_{\text{film}}\right].$$

Thus, the scattering properties of the particles are extracted from:

$$C_{\mathrm{sca}} = -\frac{V_p \ln\left(T_{\mathrm{spec}}/T_{\mathrm{tot}}\right)}{f_v t_{\mathrm{film}}}.$$

The extracted values of C_{sca} for $f_v = 0.1\%$ v/v and particles of 1 μm diameter are shown at the inset of Fig. S6(b). We fitted the results with a curve of the form $C_{\mathrm{sca}} = a/\lambda^b$, with $a = 2.4782$ and $b = 1.4095$ [Fig. S6(b)]. Finally, using Monte-Carlo simulations (see Main Text, Materials and Methods) we iterate to find the value of μ_{sca} that best matches the results of experiments. We found excellent agreement for $\mu_{\mathrm{sca}} = 0.75$ [Fig. S6(a)].

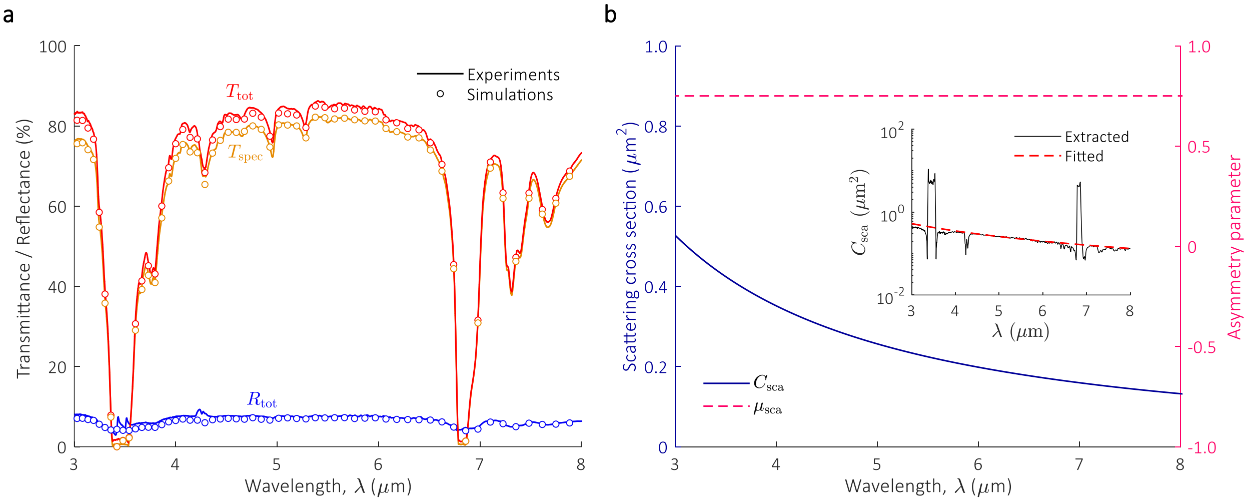

Figure S6: **Estimation of scattering properties and refractive index of the PE films used in the VO_2(M)/PE composites. (a)** The values of T_{tot}, T_{spec} and R_{tot} of a 101μm thick PE film obtained from experiments (solid lines) are compared with Monte-Carlo simulations based on the estimated refractive index and scattering properties of the PE film). **(b)** Estimated C_{sca} of the PE film for $f_v = 0.1\%$ v/v and particles of 1 μm diameter. The curve is based on a fitting curve using the extracted results from experiments (inset).

4.5 Radiative properties of VO_2(M)/PE composite films (Experiments and Simulations

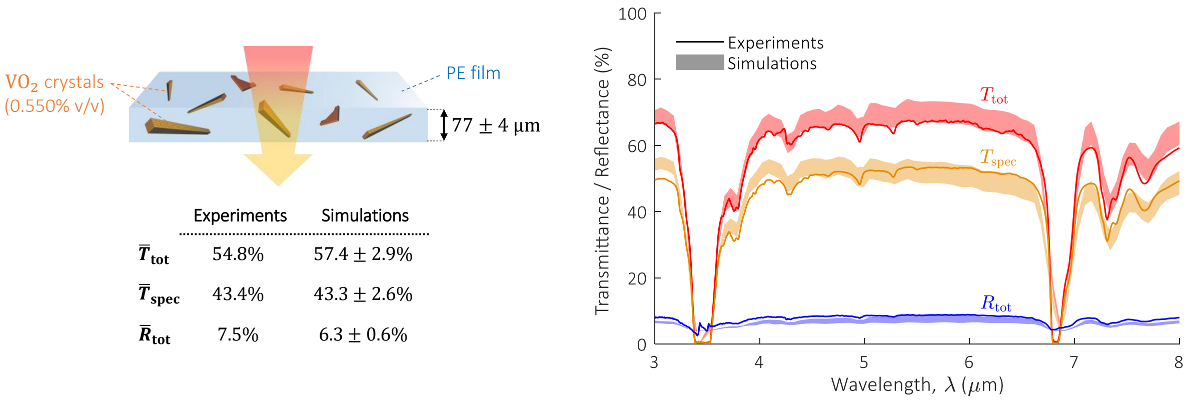

Figure S7: T_{tot}, T_{spec} **and** R_{tot} **of** VO_2**(M)/PE composite film obtained from simulation and experiments**. The composite film is composed of a PE matrix with 0.55% v/v VO_2(M) powder and has $77 \pm 4 \mu$m thickness. The scattering properties of the VO_2(M) powder are the same used in the main text [Fig. 4(a)]

5 Validation of Monte-Carlo Code

We validated our Monte-Carlo code for radiative transfer simulations against the Adding-doubling method [Fig. S8(b)]. We calculated the total transmittance (T_{tot}) and reflectance (R_{tot}), and the specular transmittance (T_{spec}) at normal incidence over a 1 mm thick film with refractive index $n_{\text{film}} = 1.4$, and 0.1% particles per volume. The particles have a diameter $D_p = 1$ μm, and the scattering properties shown in Fig. S8(a).

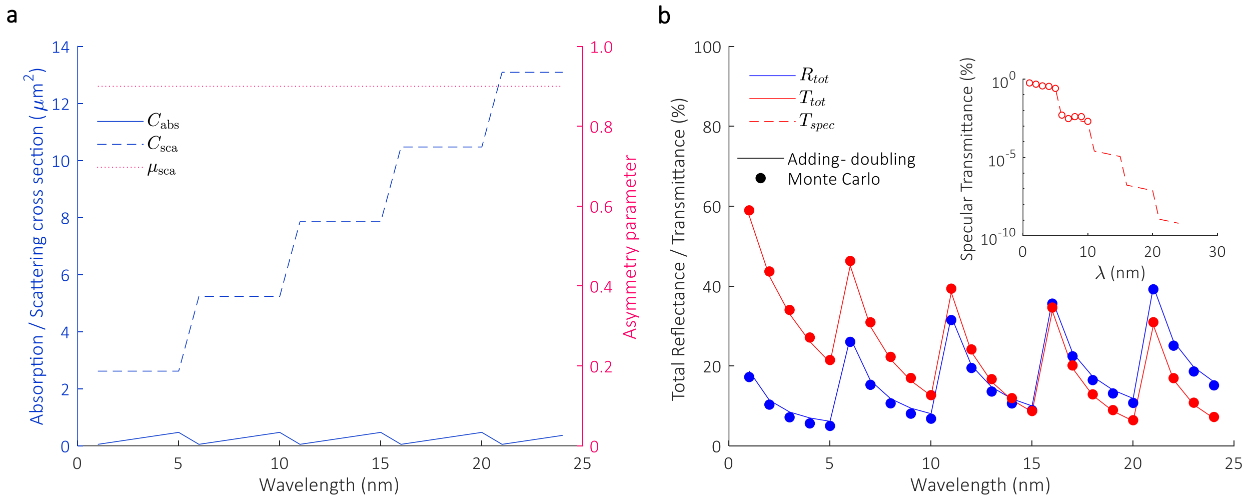

Figure S8: **Validation of Monte-Carlo code for radiative transfer calculations against Adding-doubling method**. **(a)** Light scattering properties $C_{\rm abs}$, $C_{\rm sca}$ and $\mu_{\rm sca}$ of the spherical particles (1 μm diameter) considered in this study **(b)** Total transmittance and reflectance and specular transmittance (inset) for light at normal incidence of a 1 mm thick film with 1.4 refractive index and 0.1% particles per volume. The results from our Monte-Carlo code (circles) are plotted against Adding-doubling method calculations using the code by Prahl.[30]

The results for Adding-doubling method are obtained from the open-source code by Prahl.[30] This code was used to calculate the total reflectance and transmittance. The specular transmittance is calculated as

$$T_{\rm spec} = (1 - R)\exp\left[\frac{f_v}{V_p}\left(C_{\rm abs} + C_{\rm sca}\right) t_{\rm film}\right],$$

where $R = 4.12\%$ is the reflectance of a 1 mm thick film with 1.4 refractive index, f_v is the volume fraction, V_p is the particle's volume, and $t_{\rm film}$ is the thickness of the film.

The results of total transmittance/reflectance from our Monte-Carlo code show excellent agreement with the curves from Adding-doubling method. The specular transmittance (inset of Fig. S8) shows good agreement up to 10 nm wavelength. Outside this range, the values of specular transmittance are bellow the minimum resolution of our Monte-Carlo setup (0.0001% for 1,000,000 photons).

We performed and additional test based on the total transmittance and reflectance of a

slab with particles, considering the conditions: $n_{\text{film}} = 1.0$, $t_{\text{film}} = 200\ \mu$m, $D_p = 1\ \mu$m, $f_v = 0.1\%$, $C_{\text{abs}} = 0.5236\ \mu\text{m}^2$, $C_{\text{sca}} = 4.7124\ \mu\text{m}^2$ and $\mu_{\text{sca}} = 0.75$. The results shown in Table S1, show excellent agreement against the exact solution from Van de Hulst[31] and simulations with two different Monte-Carlo codes.[32,33]

Table S1: **Validation of Monte-Carlo code for Test 1.** Total transmittance and reflectance of a slab with particles, considering the conditions: $n_{\text{film}} = 1.0$, $t_{\text{film}} = 200\ \mu$m, $D_p = 1\ \mu$m, $f_v = 0.1\%$, $C_{\text{abs}} = 0.5236\ \mu\text{m}^2$, $C_{\text{sca}} = 4.7124\ \mu\text{m}^2$ and $\mu_{\text{sca}} = 0.75$

R_{tot}	T_{tot}	Source
9.739%	66.096%	Van de Hulst[31]
9.774%	66.101%	Monte Carlo code
9.734%	66.096%	Wang, Jacques and Zheng[32]
9.711%	66.159%	Prahl et al[33]

References

(1) Krüger, M.; Bimonte, G.; Emig, T.; Kardar, M. Trace formulas for nonequilibrium Casimir interactions, heat radiation, and heat transfer for arbitrary objects. *Phys. Rev. B* **2012**, *86*, 115423.

(2) Tsang, L.; Kong, J. A.; Ding, K.-H.; Ao, C. O. *Scattering of Electromagnetic Waves: Theories and Applications*; John Wiley & Sons, Inc.: New York, USA, 2000; p 445.

(3) Molesky, S.; Jin, W.; Venkataram, P. S.; Rodriguez, A. W. T Operator Bounds on Angle-Integrated Absorption and Thermal Radiation for Arbitrary Objects. *Phys. Rev. Lett.* **2019**, *123*, 257401.

(4) Molesky, S.; Chao, P.; Jin, W.; Rodriguez, A. W. Global T operator bounds on electromagnetic scattering: Upper bounds on far-field cross sections. *Phys. Rev. Res.* **2020**, *2*, 033172.

(5) Bohren, C. F.; Huffman, D. R. *Absorption and Scattering of Light by Small Particles*; John Wiley & Sons, Inc., 1998; p 544.

(6) Chew, W. C.; Sha, W. E.; Dai, Q. I. Green's Dyadic, Spectral Function, Local Density of States, and Fluctuation Dissipation Theorem. *Prog. in Electromagn. Res.* **2019**, *166*, 147–165.

(7) Mishchenko, M. I. Radiation force caused by scattering, absorption, and emission of light by nonspherical particles. *J. Quant. Spectrosc. Radiat. Transfer* **2001**, *70*, 811–816.

(8) Polimeridis, A. G.; Reid, M. T. H.; Jin, W.; Johnson, S. G.; White, J. K.; Rodriguez, A. W. Fluctuating volume-current formulation of electromagnetic fluctuations in inhomogeneous media: Incandescence and luminescence in arbitrary geometries. *Phys. Rev. B* **2015**, *92*, 134202.

(9) Tai, C.-T. In *Dyadic Green Functions in Electromagnetic Theory*, 2nd ed.; Dudley, D. D., Ed.; IEEE Press Series: New York, USA, 1994; p 343.

(10) Rodriguez, A. W.; Reid, M. T.; Johnson, S. G. Fluctuating-surface-current formulation of radiative heat transfer: Theory and applications. *Phys. Rev. B* **2013**, *88*, 054305.

(11) Reid, M. T.; Johnson, S. G. Efficient Computation of Power, Force, and Torque in BEM Scattering Calculations. *IEEE Trans. Antennas Propag.* **2015**, *63*, 3588–3598.

(12) Ramirez-Cuevas, F. V. AVESCATTER: A SCUFF-EM application for light scatttering of randomly oriented particles of arbitrary shape. `https://github.com/PanxoPanza/scattering_random_orientation`, (Accessed: 2022-01-25).

(13) Ciesielski, A.; Skowronski, L.; Trzcinski, M.; Górecka, E.; Trautman, P.; Szoplik, T. Evidence of germanium segregation in gold thin films. *Surf. Sci.* **2018**, *674*, 73–78.

(14) Jackson, J. D. *Classical Electrodynamics*, 3rd ed.; John Wiley & Sons: New York, 1998; p 832.

(15) Suryadharma, R. N.; Rockstuhl, C. Predicting observable quantities of self-assembled metamaterials from the T-Matrix of its constituting meta-atom. *Mater.* **2018**, *11*, 213.

(16) Khlebtsov, N. G. Orientational averaging of light-scattering observables in the T-matrix approach. *Appl. Opt.* **1992**, *31*, 5359.

(17) Rahi, S. J.; Emig, T.; Graham, N.; Jaffe, R. L.; Kardar, M. Scattering theory approach to electrodynamic Casimir forces. *Phys. Rev. D* **2009**, *80*, 1–27.

(18) Golyk, V. A.; Krüger, M.; Kardar, M. Linear response relations in fluctuational electrodynamics. *Phys. Rev. B* **2013**, *88*, 155117.

(19) Mishchenko, M. I.; Hovenier, J. W.; Travis, L. D. *Light scattering by nonspherical particles: theory, measurements, and applications*, 1st ed.; Academic Press, 2000; p 720.

(20) Novotny, L.; Hecht, B. *Principles of Nano-Optics*, 1st ed.; Cambridge University Press: Cambridge, UK, 2006.

(21) Santiago, E. Y.; Besteiro, L. V.; Kong, X.-T.; Correa-Duarte, M. A.; Wang, Z.; Govorov, A. O. Efficiency of Hot-Electron Generation in Plasmonic Nanocrystals with Complex Shapes: Surface-Induced Scattering, Hot Spots, and Interband Transitions. *ACS Photonics* **2020**, *7*, 2807–2824.

(22) Muskens, O. L.; Bachelier, G.; Del Fatti, N.; Vallée, F.; Brioude, A.; Jiang, X.; Pileni, M. P. Quantitative absorption spectroscopy of a single gold nanorod. *J. Phys. Chem. C* **2008**, *112*, 8917–8921.

(23) Mkrtchian, V.; Parsegian, V. A.; Podgornik, R.; Saslow, W. M. Universal thermal radiation drag on neutral objects. *Phys. Rev. Lett.* **2003**, *91*, 220801.

(24) Mishchenko, M. I.; Travis, L. D.; Macke, A. Scattering of light by polydisperse, randomly oriented, finite circular cylinders. *Appl. Opt.* **1996**, *35*, 4927–4940.

(25) Mishchenko, M.; Travis, L.; Mackowski, D. T-Matrix Codes for Computing Electromagnetic Scattering by Nonspherical and Aggregated Particles. https://www.giss.nasa.gov/staff/mmishchenko/t_matrix.html, (Accessed: 2021-07-17).

(26) Wan, C. et al. On the Optical Properties of Thin-Film Vanadium Dioxide from the Visible to the Far Infrared. *Ann. Phys.* **2019**, *531*, 1900188.

(27) Palik, E. D.; Ghosh, G. *Handbook of optical constants of solids*; Academic Press, 1998; p 999.

(28) Katsidis, C. C.; Siapkas, D. I. General transfer-matrix method for optical multilayer systems with coherent, partially coherent, and incoherent interference. *Appl. Opt.* **2002**, *41*, 3978.

(29) Prahl, S. A.; van Gemert, M. J. C.; Welch, A. J. Determining the optical properties of turbid media by using the adding–doubling method. *Appl. Opt.* **1993**, *32*, 559–568.

(30) Prahl, S. A. Forward and Inverse Radiative Transport using the Adding-Doubling method. https://github.com/scottprahl/iad, (Accessed: 2021-03-11).

(31) Van de Hulst, H. C. *Multiple Light Scattering*, 1st ed.; Academic Press: New York, USA, 1980; Vol. II; p 332.

(32) Jacques, S. L.; Wang, L. MCML—Monte Carlo modeling of light transport in multi-layered tissues. *Comput. Methods Programs Biomed.* **1995**, *47*, 131–146.

(33) Prahl, S. A. A Monte Carlo model of light propagation in tissue. *Proc. SPIE, Dosimetry of Laser Radiation in Medicine and Biology* **1989**, *10305*, 102 – 111.
